# Escherichia coli Leucine-Responsive Regulatory Protein Bridges DNA *In Vivo* and Tunably Dissociates in the Presence of Exogenous Leucine

**DOI:** 10.1128/mbio.02690-22

**Published:** 2023-02-14

**Authors:** Christine A. Ziegler, Lydia Freddolino

**Affiliations:** a Department of Biological Chemistry, University of Michigan Medical School, Ann Arbor, Michigan, USA; b Department of Computational Medicine and Bioinformatics, University of Michigan Medical School, Ann Arbor, Michigan, USA; Washington University in St. Louis School of Medicine

**Keywords:** DNA bridging, FFRP, global regulator, Lrp, transcriptional regulation

## Abstract

Feast-famine response proteins are a widely conserved class of global regulators in prokaryotes, the most highly studied of which is the Escherichia coli leucine-responsive regulatory protein (Lrp). Lrp senses the environmental nutrition status and subsequently regulates up to one-third of the genes in E. coli, either directly or indirectly. Lrp exists predominantly as octamers and hexadecamers (16mers), where leucine is believed to shift the equilibrium toward the octameric state. In this study, we analyzed the effects of three oligomerization state mutants of Lrp in terms of their ability to bind to DNA and regulate gene expression in response to exogenous leucine. We find that oligomerization beyond dimers is required for Lrp’s regulatory activity and that, contrary to previous speculation, exogenous leucine modulates Lrp activity at its target promoters exclusively by inhibiting Lrp binding to DNA. We also show evidence that Lrp binding bridges DNA over length scales of multiple kilobases, revealing a new range of mechanisms for Lrp-mediated transcriptional regulation.

## INTRODUCTION

Bacterial transcription is controlled in a hierarchical manner in which a small number of highly expressed global regulators exert control over the expression of hundreds of transcription factors and downstream genes (1, 2). Escherichia coli has seven classically recognized global regulators: CRP, IHF, FNR, Fis, ArcA, H-NS, and Lrp (leucine-responsive regulatory protein) (1). Lrp is of particular interest due to its crucial role in nutrient sensing and its high level of conservation among prokaryotes; nearly half of sequenced bacteria and almost all sequenced archaea contain one or more Lrp homologs (3). While Lrp was originally identified in 1973 (4) and has since been shown to regulate as many as 32% of E. coli genes either directly or indirectly (5), little is known about the precise mechanism by which Lrp regulates its target genes (6).

Lrp is a small (18.8-kDa), basic (pI = 9.2), and highly abundant (~3,000 dimers/cell) DNA binding protein in E. coli (7) and is thought to sense the environmental nutritional status via exogenous l-leucine and regulate gene expression accordingly (7). The E. coli Lrp regulon consists of genes involved in amino acid metabolism, one-carbon metabolism, peptide and amino acid transport, flagellum and fimbria biosynthesis, osmotic stress, acid stress, and other virulence factors (5, 8–10), and Lrp is known to regulate 70% of the genes involved in the transition from logarithmic growth to stationary phase as nutrients are depleted (11). To accomplish its regulatory activities, Lrp was observed to utilize six modes of gene regulation: Lrp can activate or repress its target genes, and for each target, leucine can have no effect, augment Lrp’s effect, or inhibit Lrp (8, 9). Through several *in vitro* studies, it was hypothesized that Lrp exists predominantly as a hexadecamer (16mer) when l-leucine concentrations are low (such as in minimal medium) but that the oligomeric state switches to predominantly Lrp octamers in the presence of l-leucine, suggesting that leucine modulates Lrp function by altering Lrp’s oligomerization state (12–14). However, this hypothesis does not fully explain how leucine can augment Lrp function at some promoters, have no effect at others, and somehow inhibit the rest.

In this study, we determined a more precise mechanism by which exogenous l-leucine modulates Lrp gene regulation by analyzing the global effects of leucine on Lrp binding to DNA and its subsequent effects on gene expression via paired Lrp chromatin immunoprecipitation sequencing (ChIP-seq) and RNA polymerase (RNAP) ChIP-seq. In order to further investigate the function of various Lrp oligomerization states, we also analyzed the effects of exogenous leucine on a “dimer-only” Lrp mutant (ΔC11) and two “octamer-only” Lrp mutants, D114E and L136R, which were originally discovered through their inability to respond to leucine at the *ilvIH* promoter and were later characterized as octamer-only mutants through size exclusion chromatography and dynamic light scattering (12–15). Through these high-throughput experiments and targeted follow-up case studies, we determined that exogenous l-leucine inhibits Lrp binding to DNA at its target promoters. Lrp likely exists in an equilibrium of various higher-order oligomeric complexes that bridge DNA in minimal medium depending on the DNA sequence, and only the strongest Lrp binding sites maintain Lrp bound to the DNA in the presence of l-leucine. The dimer-only mutant is unable to bind DNA under any condition tested, demonstrating that Lrp requires oligomers larger than a dimer in order to bind DNA. The Lrp L136R mutant is mostly unable to sense exogenous leucine and therefore maintains higher-order oligomeric nucleoprotein complexes on target promoters, even in the presence of leucine. Meanwhile, the D114E mutant binds to canonical Lrp sites as well as additional nearby sites in minimal medium, suggesting that D114E stabilizes higher-order Lrp oligomers that bridge DNA. Thus, while neither the L136R nor the D114E mutant is strictly “octamer only,” the differing effects of these mutations on Lrp’s DNA binding and regulatory output allow us to parse out the effects of ligand binding and oligomerization.

## RESULTS

### Oligomeric Lrp mutants show altered growth phenotypes and regulatory outputs.

**FIG 1:**
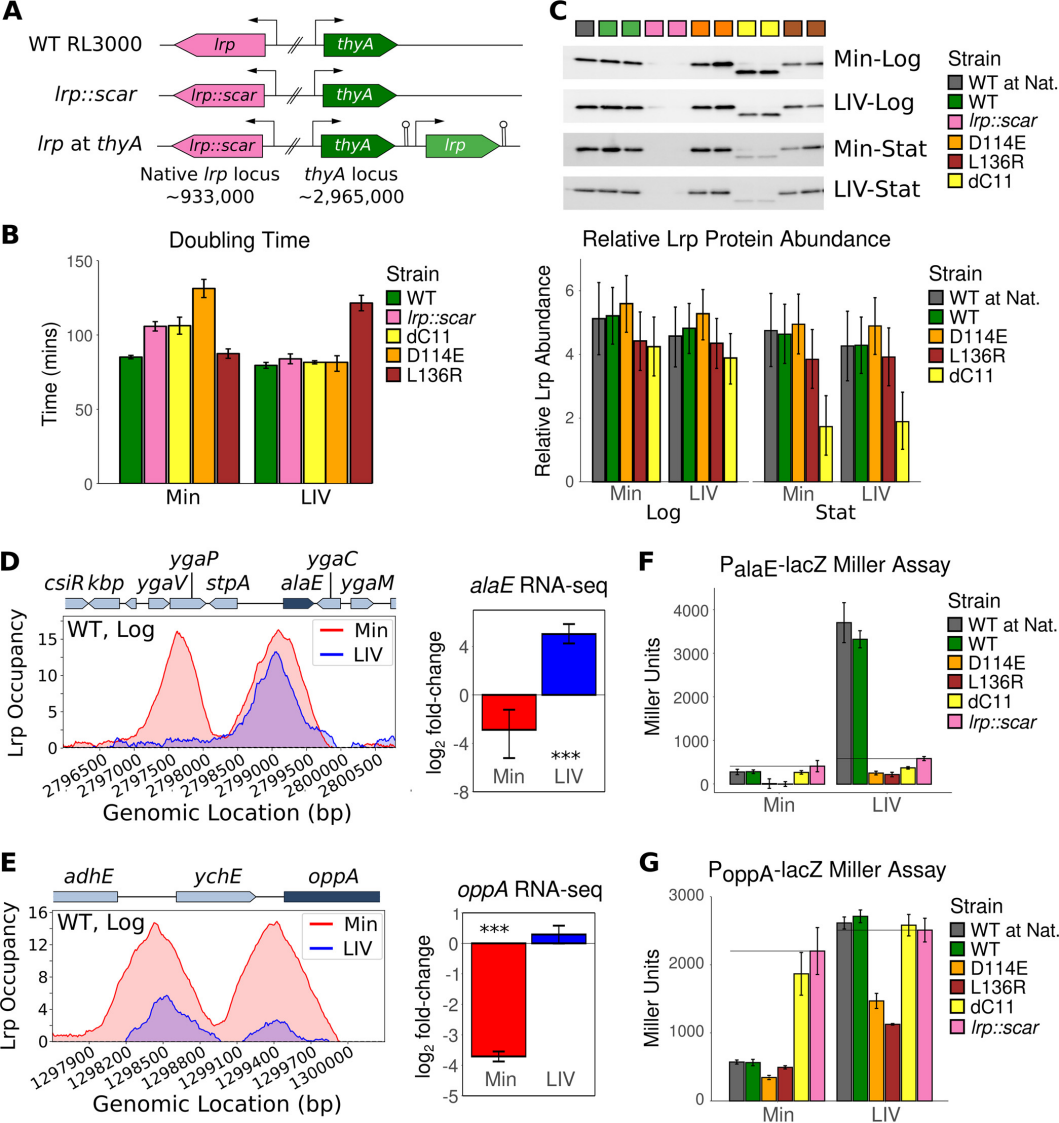
Lrp mutants display unique and consistent phenotypes. (A) Schematic of *lrp* genotypes used in our study. WT RL3000 contains *lrp* at the native locus (pink), whereas in the *lrp*::*scar* and *lrp* at *thyA* strains, the *lrp* ORF at the native locus has been fully removed. The *thyA* locus in these strains contains either *thyA* alone (*lrp*::*scar*) or *thyA* immediately upstream of a bidirectional-terminator-flanked *lrp* gene with its native promoter (*lrp* at *thyA*). Arrows represent promoters, hairpins represent bidirectional terminators, and numbers below the genes indicate their approximate genomic coordinates. All strains were constructed in duplicate; we refer to the two copies of each intended genotype as “lineage replicates.” (B) The doubling times of *lrp* strains in Min and LIV M9 minimal media were calculated during mid-log phase. Each bar represents the mean from two biological replicates each of two independently constructed lineages of each strain. Error bars represent the standard deviations across all four observations. All *lrp* variants in this experiment are at the *thyA* locus, including the WT. (C, top) Representative Western blot for each condition and time point showing both lineages of each strain (see Text S3 in the supplemental material for details). (Bottom) Inferred Lrp abundances under each condition obtained by fitting a Bayesian model for Lrp protein levels (based on the pixel density of Western blots divided by the sum of the pixel density from a total protein blot) across strains, conditions, and both biological and lineage replicates. Error bars indicate 95% credible intervals. For model fitting, the *lrp*::*scar* strain was excluded because there was no Lrp present in the Western blots for these samples. (D, left) Replotted WT Lrp ChIP-seq data from a previous study (5) for the *stpA-alaE* region during mid-log phase, where the *y* axis represents the *lrp*::*scar*-subtracted z-scores of Lrp-ChIP relative the to input for Min (red) and LIV (blue). (Right) Replotted log_2_ fold changes of *alaE* transcript levels from a previous study (5) of WT Lrp cells relative to *lrp*::*scar* cells during mid-log phase in Min (red) and LIV (blue). Error bars represent 95% confidence intervals. (E) Same as panel D except for the *oppA* locus. (F) Bar graph representing the mean Miller units from the *P_alaE_*-*lacZ* reporter Miller assay from two technical replicates from each of two lineage replicates for each strain under each condition (Min and LIV) at mid-log phase. Error bars represent the standard deviations across all four observations. Black horizontal lines represent the Miller units for the *lrp*::*scar* strain under that condition. (G) Same as panel F except with the *P_oppA_-lacZ* reporter construct.

After constructing a reliable system for introducing and genetically modifying *lrp* at the *thyA* locus (Fig. 1A; see also Table S1 and Text S1 in the supplemental material) (28, 29), we proceeded to investigate how wild-type (WT) Lrp and various Lrp oligomerization mutants (D114E, L136R, and ΔC11) affected the general physiology of E. coli cells in minimal medium (Min) and the same medium supplemented with branched-chain amino acids (leucine/isoleucine/valine [LIV], in either exponentially growing (Log) or stationary phase (Stat) cells). The strain harboring the dimer-only ΔC11 mutant had roughly the same growth rate as that of the *lrp*::*scar* (“scar” refers to the FRT recombinase site left after marker excision) strain under both conditions (Fig. 1B). Intriguingly, while D114E and L136R were both originally identified as octamer-only mutants through size exclusion chromatography and dynamic light scattering experiments and were thus expected to behave similarly (13), D114E had a strong growth defect in Min that was restored in LIV, whereas L136R had the opposite effect on the growth rate (Fig. 1B). We next investigated the Lrp protein levels in each of our strains via Western blotting and confirmed that WT Lrp expressed from the *thyA* locus was present at levels comparable to those of WT Lrp expressed from the native *lrp* locus (Fig. 1C and Table S2). Despite ΔC11 strains growing at rates comparable to those of the *lrp*::*scar* strain, the dimer-only ΔC11 protein is present at only slightly lower levels than those of WT Lrp during logarithmic growth (>97% posterior probability of being lower than the WT level based on our Bayesian analysis [here, we abbreviate the posterior probability of a difference in the observed direction as *P*_diff_]); however, ΔC11 levels are substantially reduced over the course of the growth curve (*P*_diff_ > 99%). We obtained weaker evidence that D114E Lrp was more highly expressed than WT Lrp under all conditions tested (*P*_diff_ > 76%), while L136R Lrp was expressed at lower levels than WT Lrp (*P*_diff_ > 80%) (Fig. 1C and Table S2), which may partially explain the differential growth rates of these two octamer-only mutants. Thus, contrary to previous expectations regarding the octamer-only D114E and L136R Lrp variants, they in fact appear to have distinct responses to changing nutrient conditions.

10.1128/mbio.02690-22.1 TEXT S1 Description of the genetic background used for *lrp* mutants in the present study. Download Text S1, PDF file, 0.04 MB. Copyright © 2023 Ziegler and Freddolino. 2023 Ziegler and Freddolino. https://creativecommons.org/licenses/by/4.0/ This content is distributed under the terms of the Creative Commons Attribution 4.0 International license.

10.1128/mbio.02690-22.7 TABLE S1 Strains used in the present study. Shown for each strain are the name commonly used in the present work, the complete genotype, and origins (see Text S3 in the supplemental material for details of strain construction). Download Table S1, XLSX file, 0.01 MB. Copyright © 2023 Ziegler and Freddolino. 2023 Ziegler and Freddolino. https://creativecommons.org/licenses/by/4.0/ This content is distributed under the terms of the Creative Commons Attribution 4.0 International license.

10.1128/mbio.02690-22.3 TEXT S3 Detailed description of all methods used in the present study. Download Text S3, PDF file, 0.1 MB. Copyright © 2023 Ziegler and Freddolino. 2023 Ziegler and Freddolino. https://creativecommons.org/licenses/by/4.0/ This content is distributed under the terms of the Creative Commons Attribution 4.0 International license.

10.1128/mbio.02690-22.8 TABLE S2 Statistical analysis of quantitative Western blot data. Summary statistics are given for Bayesian fits to the quantitative Western blot data shown in Fig. 1C. “value” indicates the fitted value from the model, “ci.lo” and “ci.hi” are the bottom and top of a 95% credible interval for the value, “P_diff” indicates the posterior probability of a difference versus the wild-type strain in the same medium in the indicated direction, and “fitted_plot” and subsequent columns indicate the fitted values (and credible intervals) transformed back to the original intensity units. Download Table S2, XLSX file, 0.01 MB. Copyright © 2023 Ziegler and Freddolino. 2023 Ziegler and Freddolino. https://creativecommons.org/licenses/by/4.0/ This content is distributed under the terms of the Creative Commons Attribution 4.0 International license.

To assess how the oligomerization Lrp mutants affect gene expression at well-studied Lrp-leucine regulated promoters, we next constructed *lacZ* reporter strains at the native *alaE* and *oppA* promoters. These two loci were selected due to their unique Lrp binding patterns and responses to leucine in previous experiments (5). The *alaE* promoter contains two strong Lrp peaks in Min that cause a slight repression of *alaE*, whereas only one of the two Lrp peaks is maintained in LIV, causing the strong activation of *alaE* (Fig. 1D). On the other hand, WT Lrp forms two strong peaks at the *oppA* promoter in Min that repress *oppA*, but these Lrp peaks are both abrogated in LIV, and *oppA* becomes derepressed (Fig. 1E). Our WT and *lrp*::*scar* Miller assays reproduced these findings reported previously by Kroner et al. (5) for both *alaE* (Fig. 1F) and *oppA* (Fig. 1G). Furthermore, the regulation at both of these promoters by WT Lrp expressed from either the native locus (Fig. 1, black bars) or the *thyA* locus (green bars) was comparable across conditions, confirming that our *lrp-thyA* constructs mimic WT strains. The dimer-only Lrp mutant (ΔC11) behaved like the *lrp::scar* mutant at *oppA* (Fig. 1G) but appeared to have a slight repressive effect on *alaE* relative to *lrp*::*scar* regardless of the LIV status (Fig. 1F), suggesting that Lrp ΔC11 has a possible regulatory effect on at least some Lrp-regulated promoters.

Both D114E Lrp and L136R Lrp repressed *oppA* in Min and maintained most of their repression in LIV (Fig. 1G). These two octamer-only Lrp mutants also behaved similarly at the *alaE* promoter, where they both repressed *alaE* even more strongly than WT Lrp in minimal medium, and this repression was relieved in LIV (Fig. 1F). Unlike WT Lrp, for both octamer-only mutants, *alaE* expression was not activated by LIV. Collectively, these data confirm that our genetic system reproduces previous data and also demonstrate the potential regulatory role of dimer-only ΔC11. However, at least for the two target promoters considered here, the octamer-only D114E and L136R variants have similar regulatory effects, which cannot explain the opposite growth phenotypes of these two strains (Fig. 1B). To address the discrepancy between the observed results of reporter assays and the growth phenotypes, we took a high-throughput approach to globally assess Lrp binding and regulation of gene expression in response to different Lrp mutants and conditions.

### Paired Lrp and RNAP ChIP-seq reproduces previous findings and reveals unique binding patterns for each oligomerization Lrp mutant.

**FIG 2:**
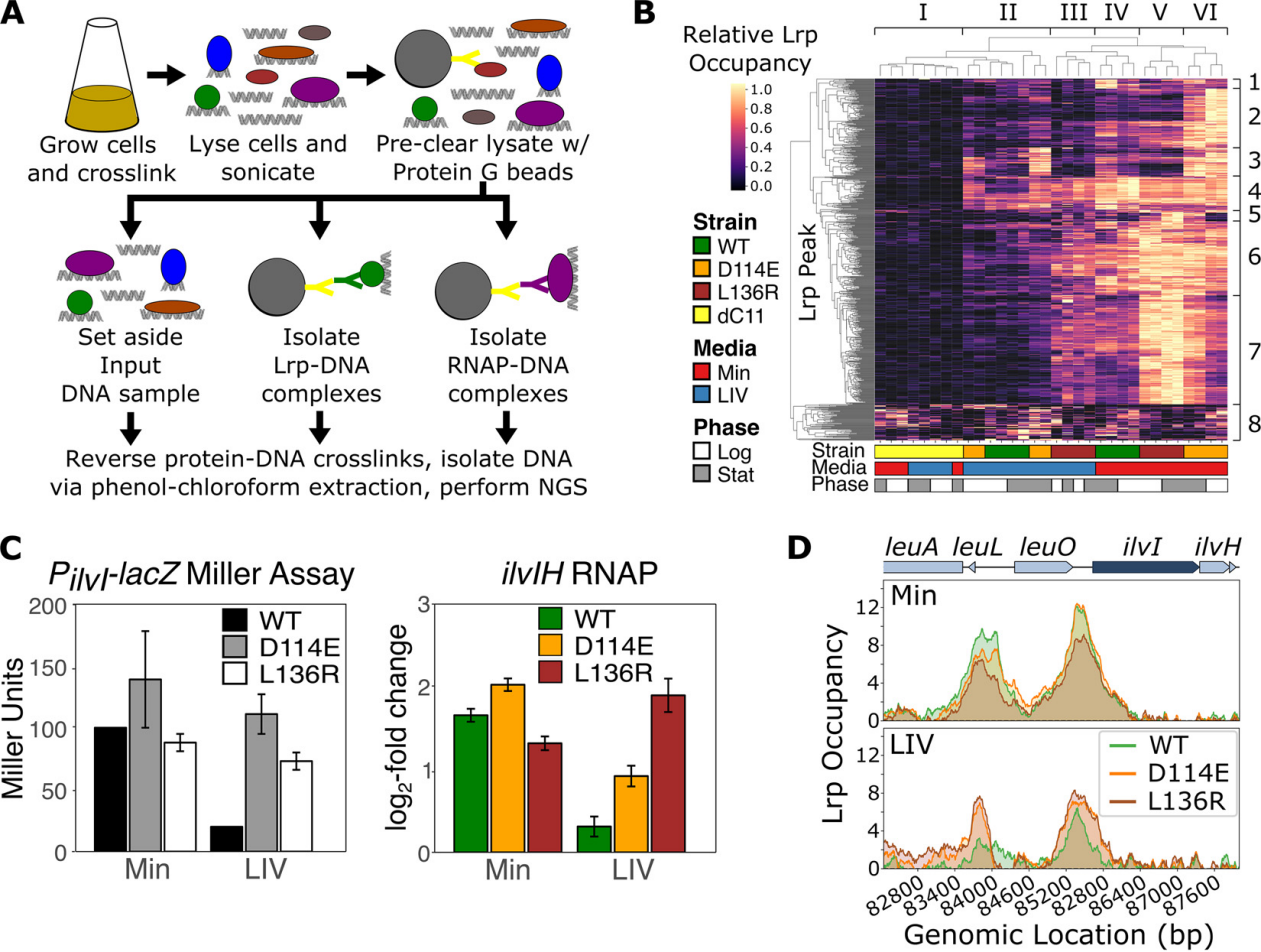
Paired Lrp and RNAP ChIP-seq reveals several key features of Lrp binding and regulatory effects. (A) Schematic of the paired Lrp and RNAP ChIP-seq experimental workflow, where proteins are represented as colored circles; magnetic protein G beads are represented as large gray circles with a Y-shaped yellow antibody attached to the surface; Lrp and RNAP are represented by green and purple circles, respectively; and Lrp- and RNAP-specific antibodies are represented by green and purple Y shapes, respectively. See Text S3 in the supplemental material for a detailed protocol. NGS, next-generation sequencing. (B) Heat map of Lrp occupancy at all Lrp binding regions across all strains, media, and time points, where the *x* axis indicates each sample, the *y* axis represents each of the 793 significant unique Lrp binding sites that were identified in at least one strain under at least one condition, and the color at each position represents the row-normalized Lrp occupancy in that sample at that location (Lrp occupancy divided by the maximum value in each row). Each column represents the mean from two biological replicates, and each lineage replicate is plotted separately. Both the *x* and *y* axes were clustered by Euclidean distance (taking the average distances between cluster members), generating Groups I to VI (samples) on the *x* axis and Classes 1 to 8 (binding-site categories) on the *y* axis. (C) Comparison of replotted data from *P_ilvIH_*-*lacZ* Miller assays reported previously by Platko and Calvo (15) for the Lrp variants shown (left) with our RNAP ChIP-seq data for *ilvIH* (right). The RNAP ChIP data show the log_2_ fold changes in *lrp*::*scar*-subtracted RNAP occupancy relative to the input for *ilvIH* for WT, D114E, and L136R Lrp at mid-log phase in Min and LIV. Each bar represents the mean from two biological replicates for each of the two lineages, and the error bars represent the standard errors. (D) Mean Lrp occupancy (*lrp*::*scar*-subtracted) robust z scored log-ratio (rz-log ratio of Lrp relative to the input) (*y* axis) across both biological and lineage replicates for each strain in Min (top) and LIV (bottom) during mid-log phase at the *ilvIH* locus. Changes in RNAP occupancy in panel C are roughly correlated with Lrp occupancy upstream of *ilvIH*.

We performed a paired Lrp and RNA polymerase (RNAP) ChIP-seq experiment (Fig. 2A) to measure global Lrp binding and regulatory effects and detected a total of 793 significant Lrp binding sites across the genome under at least one condition. The samples (Fig. 2B, *x* axis) are clustered into six groups (I to VI). Group I consists of dimer-only Lrp ΔC11 samples and generally exhibits low levels of binding to DNA at most Lrp binding sites, indicating that this dimer-only mutant is deficient in DNA binding. We thus excluded Lrp ΔC11 from further ChIP-seq analyses. Group II is a mixture of WT and D114E samples, both grown in LIV, which also exhibited lower levels of Lrp binding, with the exception of a few highly occupied sites. Group III (L136R-LIV) and Group IV (WT-Min) cluster closely, indicating that at the genome-wide level, L136R often fails to recognize exogenous leucine. Similarly, Group V (L136R-Min) and Group VI (D114E-Min) cluster closely and exhibit the highest levels of Lrp binding of all samples. Given that D114E and L136R were both identified as octamer-only mutants, it was notable that D114E exhibits exogenous leucine sensitivity but that L136R does not (given the similarity of Groups III and IV), potentially explaining the difference in the growth rates between these two mutants (Fig. 1B). As described in Text S2, we also categorized the genes into eight classes. Overall, the general trend is that Lrp is more broadly bound to DNA in minimal medium than in leucine-containing medium (with the exception of L136R, which mostly cannot sense leucine). Furthermore, there are many novel sites occupied by D114E and, to a much lesser extent, L136R. Thus, a genome-wide perspective reveals substantive differences in the behaviors of the two octamer-only (D114E and L136R) mutants, with D114E showing a fundamentally altered profile of binding locations, whereas L136R binds more strongly to canonical Lrp sites in the genome and persists in binding regardless of leucine.

10.1128/mbio.02690-22.2 TEXT S2 Detailed description of different classes of Lrp-regulated genes. Download Text S2, PDF file, 0.03 MB. Copyright © 2023 Ziegler and Freddolino. 2023 Ziegler and Freddolino. https://creativecommons.org/licenses/by/4.0/ This content is distributed under the terms of the Creative Commons Attribution 4.0 International license.

To quantify differences in Lrp binding across genotypes and conditions, we first calculated the number of significant peaks, both under each condition and overall within a genotype (Fig. S1). Overall, WT Lrp had the fewest peaks (440 overall), with L136R and D114E having significantly more peaks (517 and 556, respectively). However, the overall distribution of peaks within a genotype across conditions remained similar, with Min-Log and Min-Stat having the most peaks and LIV-Log having the fewest. We next investigated the role of these Lrp peaks in gene regulation and saw that Lrp binding sites are strongly enriched at promoters (Fig. S1), with 37.95% of WT, 35.07% of D114E, and 35.98% of L136R peaks overlapping at least one annotated transcription start site (TSS), all representing significant enrichments over what would be expected by chance (*P* < 0.01 by approximate permutation tests). For the Lrp peaks overlapping a TSS, we next calculated the number of transcription units (TUs) that are directly regulated by Lrp (i.e., Lrp binds to the TSS, and there is a significant Lrp-dependent change in RNAP occupancy across the gene body) and found that 41.92% of WT-, 33.33% of D114E-, and 51.61% of L136R-regulated TUs were under direct Lrp regulation under at least one condition tested. Given that Lrp binding is strongly enriched at promoters, it is likely that the remaining poised Lrp sites could become direct targets under a condition not tested in this experiment, as was previously suggested (5). The role of Lrp sites within open reading frames (ORFs) remains unclear, although given that the presence of Lrp at these sites does not significantly affect the expression of the respective genes, it is likely that these sites serve as decoys in order to correctly titrate Lrp levels at functional sites (16, 17).

10.1128/mbio.02690-22.4 FIG S1 Heat maps of total Lrp peaks, Lrp peaks at promoters, and direct Lrp targets for each strain and condition. For each plot, the numbers in each box represent the total numbers (or percentages) of unique Lrp peaks or transcription units (TUs) across both biological replicates of both lineage replicates for each strain under each condition. The “Total” column in each heat map represents the total number of unique Lrp peaks or TUs within each strain across all conditions and is not simply the sum across the row. Asterisks represent the significance relative to WT Lrp under that condition (*, *P* < 0.05; **, *P* < 0.01; ***, *P* < 0.001). The significance for total numbers was calculated by comparing rates using the poisson.test function in R, whereas the significance of percentages was determined using a 2-sample test for the equality of proportions using the prop.test function in R. Download FIG S1, TIF file, 0.9 MB. Copyright © 2023 Ziegler and Freddolino. 2023 Ziegler and Freddolino. https://creativecommons.org/licenses/by/4.0/ This content is distributed under the terms of the Creative Commons Attribution 4.0 International license.

To further validate our system and confirm that the high-throughput paired Lrp and RNAP ChIP-seq data reflected previous findings, we examined the regulation of the *ilvIH* locus. In their 1993 study using *P_ilvIH_-lacZ* reporters, Platko and Calvo found that WT Lrp (compared with a Δ*lrp* strain) strongly activated *ilvIH* in Min but not LIV, whereas D114E and L136R strongly activated *ilvIH* in both Min and LIV (replotted in Fig. 2C, left) (15). We note that D114E and L136R were first identified for their inability to respond to leucine at the *ilvIH* promoter. The change in the average RNAP occupancy (relative to that of the *lrp*::*scar* mutant) across the *ilvIH* transcripts in our experiments (Fig. 2C, right) revealed the same patterns of Lrp regulation, demonstrating strong reproducibility between our experiments. Furthermore, we noticed a striking correlation between the degree of Lrp binding at the *ilvIH* promoter and *ilvIH* expression across strains and media, where LIV reduced WT Lrp binding to the promoter but not D114E or L136R (Fig. 2D).

### Leucine inhibits WT Lrp binding to DNA.

**FIG 3:**
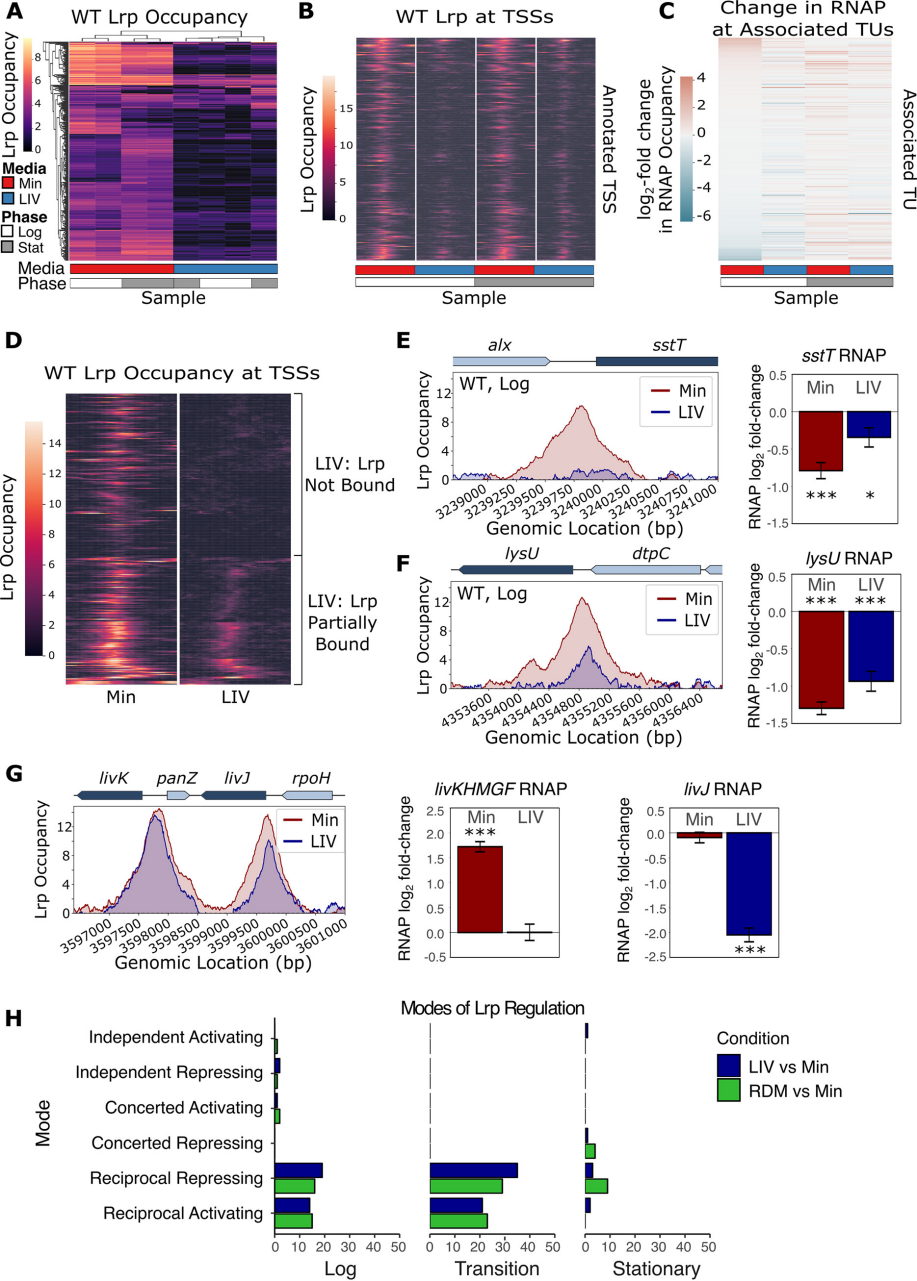
Leucine abrogates or reduces WT Lrp binding to DNA. (A) Heat map of WT Lrp occupancy at all significant WT Lrp binding sites across conditions. The *y* axis contains all 440 significant WT Lrp binding sites, the *x* axis shows the averages from both biological replicates for each lineage replicate in each medium (red, Min; blue, LIV) and each time point (white, mid-log phase; gray, stationary phase), and the color at each location represents the *lrp*::*scar*-subtracted Lrp occupancy. Both the *x* and *y* axes are clustered by similarity. Note that because of our use of lineage replicates, in this and subsequent figures using similar formats, each combination of biological variables (i.e., medium, genotype, and growth phase) appears twice, with each instance representing the average across biological replicates for a given lineage. (B) Transcription start site (TSS) pileup plot for each WT Lrp condition. TSS pileup plots, where each row represents a Lrp-regulated TSS and each column is centered on the annotated TSS, show the WT Lrp occupancy within ±1 kb of that TSS (color), oriented so that transcription proceeds to the right. For each of the four conditions (columns), the WT Lrp occupancy shown represents the mean from both biological replicates of both lineage replicates. (C) Heat map of changes in RNAP occupancy showing the log_2_ fold change in the *lrp*::*scar*-subtracted RNAP signal relative to the input for the transcription unit (TU) downstream of the annotated TSS in the corresponding row of the pileup plot from panel B, where a positive value (red) indicates activation and a negative value (blue) indicates repression. The color represents the mean change in RNAP occupancy across the entire TU for both biological replicates of both lineage replicates. (D) TSS pileup plot for WT Lrp in mid-log phase (equivalent to the first two columns of panel B), sorted by the effect of LIV on Lrp binding at each TSS. The data fall into two distinct classes of WT Lrp binding at a TSS: leucine fully abrogates WT Lrp binding (top), or leucine reduces WT Lrp binding to the TSS (bottom). (E) Example of LIV abrogating WT Lrp binding to a TSS. (Left) WT Lrp occupancy at the *sstT* locus at mid-log phase in Min (red) or LIV (blue). (Right) Log_2_ fold change in RNAP occupancy across the *sstT* TU at mid-log phase in Min (red) or LIV (blue), where positive values represent gene activation and negative values represent repression. Error bars represent the standard errors of the means. *, independent hypothesis weighting (IHW) *q* value of <0.05; **, IHW *q* value of <0.01; ***, IHW *q* value of <0.001. (F) Example of LIV significantly reducing WT Lrp binding to a TSS (same as panel E except at the *lysU* locus). (G) Example of LIV having a minimal effect on WT Lrp binding to a TSS (same as panel E except at the *livKHMGF-livJ* locus). (H) Classification of TUs into the six modes of gene regulation based on RNA expression changes and Lrp binding to the promoter regions. Direct Lrp targets from the paired Lrp ChIP-seq and RNA-seq data set reported previously by Kroner et al. (5) were classified into one of the six categories of Lrp gene regulation (independent, concerted, or reciprocal with Lrp either activating or repressing) based on whether Lrp was bound to the associated TSS and the changes in gene expression were significant. Because that study used Min, LIV, and rich defined medium (RDM), all genes were classified into the six classes of Lrp regulation by comparing either LIV to Min (blue) or RDM to Min (green). “Transition” indicates the time point when cells are transitioning from logarithmic growth into stationary-phase growth.

One of the major questions regarding E. coli Lrp is how leucine can have no effect on Lrp at some promoters (independent), a concerted effect with Lrp on others, and an inhibitory (reciprocal) effect on Lrp for the remainder (8, 9). To globally address this question, we first plotted Lrp occupancy within each sample at all 440 significant WT Lrp binding sites (Fig. 3A). It is immediately apparent that leucine reduces or completely prevents Lrp binding at nearly all of these regions, suggesting that exogenous leucine ultimately acts to inhibit Lrp binding to DNA. To investigate the relationship between WT Lrp binding at promoters and the changes in expression at the accompanying TUs, we next plotted the occupancy of Lrp within a 2-kb window of all 142 annotated TSSs overlapped by a WT Lrp peak (Fig. 3B) alongside the Lrp-dependent change in RNAP occupancy at the associated TU (here, we use the presence of RNA polymerase in the gene body as a proxy for active transcription and quantify it accordingly) (Fig. 3C). The LIV-Log condition in Fig. 3B shows greatly reduced Lrp occupancy at TSSs relative to Min-Log, and these changes in Lrp binding at TSSs are associated with substantial changes in gene expression (Fig. 3C [note that the RNAP occupancy at promoters shows substantially different rank orderings in LIV-Log versus Min-Log]).

We noticed that the TSSs with the highest WT Lrp occupancy in LIV were those with the greatest Lrp-mediated changes in expression at the associated TU (Fig. 3B, top and bottom rows of the LIV-Log column). To further investigate the effect of exogenous leucine on Lrp binding to DNA, we next clustered the WT Lrp-regulated TSSs by similarity in Lrp occupancy profiles during exponential growth. These TSSs fell into two distinct classes: those that completely lost Lrp binding in LIV relative to Min (Fig. 3D, top) and those that were partially bound in LIV relative to Min (Fig. 3D, bottom). As an example of the former case, Fig. 3E illustrates WT Lrp occupancy at the *sstT* locus, where Lrp binding is abrogated in LIV. The removal of Lrp binding to the *sstT* promoter in LIV also caused reduced repression of *sstT* (Fig. 3E, right, where Lrp-dependent repression is reduced in LIV). In contrast, Fig. 3F shows WT Lrp occupancy at the *lysU* locus, where Lrp occupancy is reduced but not completely abrogated in LIV relative to Min; here, the change in *lysU* expression between Min and LIV is not as strong as that for *sstT* (Fig. 3F, right). Out of all 142 WT Lrp-regulated TUs, there was only one exception to the trend that leucine inhibits or reduces Lrp binding to DNA, *livK*-*livJ* (Fig. 3G), which maintained high Lrp occupancy in LIV albeit still slightly reduced relative to that in Min. Intriguingly, despite *livKHMGF* and *livJ* having similar Lrp occupancies at their TSSs in Min and LIV, the Lrp-dependent expression levels are significantly different between these two conditions, indicating that other factors are interacting at these promoters with Lrp, as has also been observed for the *pap* promoter in uropathogenic E. coli (18).

Collectively, our findings suggest that the role of leucine in Lrp-mediated gene regulation is to inhibit Lrp binding to DNA (either partially or fully, depending on the site). To address the discrepancy with previous classifications of Lrp targets into concerted, reciprocal, or independent (see above), we turned to the paired Lrp ChIP-seq and RNA sequencing (RNA-seq) data set reported previously by Kroner et al. (5) and classified all directly Lrp-regulated TUs (defined as those with Lrp bound at the promoter that also had significant Lrp-dependent changes in expression under at least one condition) into one of the six previously posited modes of regulation (Fig. 3H). The only categories containing substantial numbers of TUs were the reciprocal activating and reciprocal repressing modes, further demonstrating that leucine’s role in Lrp gene regulation is almost exclusively to inhibit Lrp and that any instances of the other four modes of regulation represent highly unusual special cases (and may well arise partly due to indirect effects of Lrp).

### L136R Lrp shows greatly reduced sensitivity to exogenous leucine.

**FIG 4:**
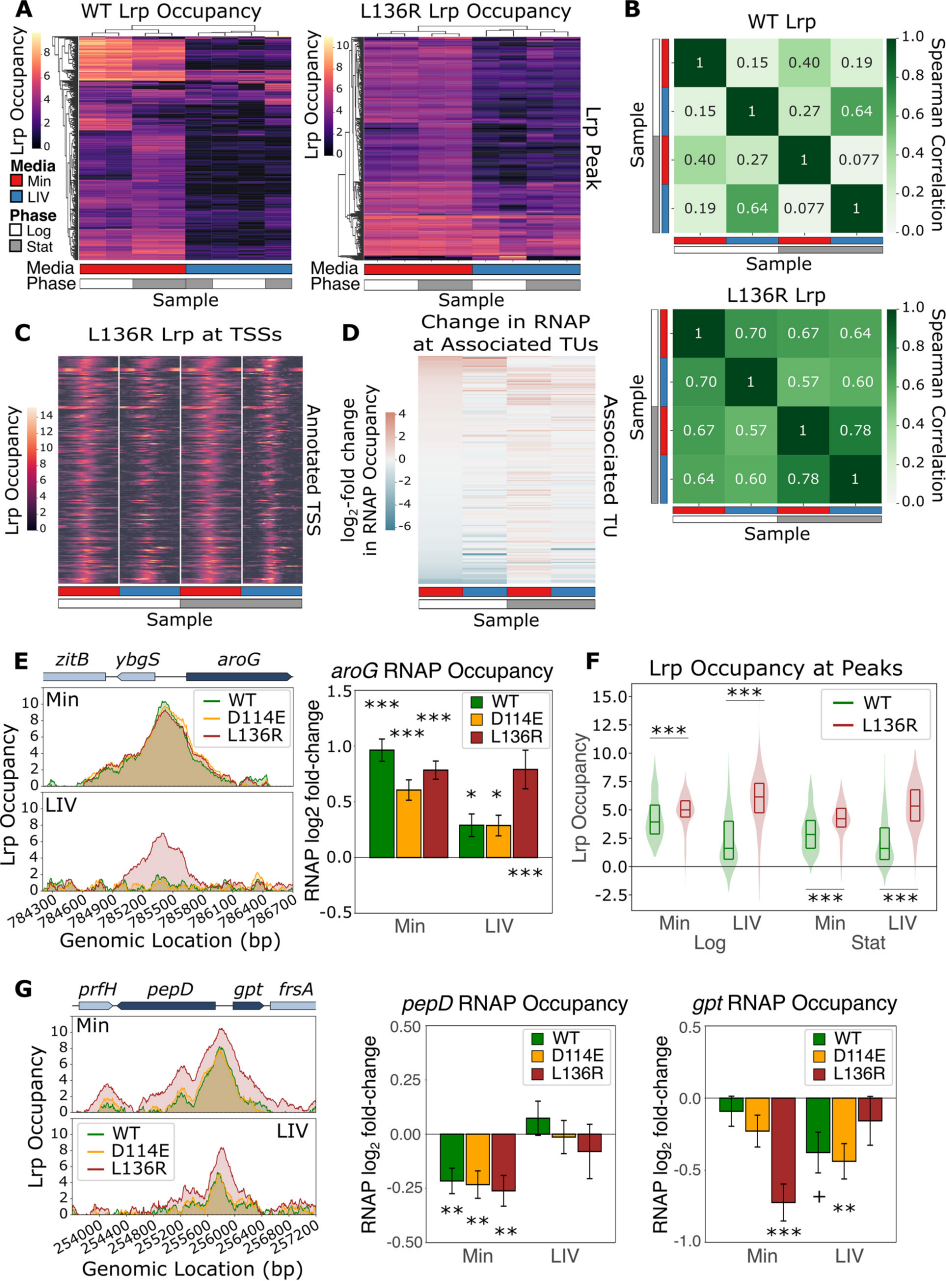
L136R binds more strongly to WT Lrp DNA binding sites, regardless of LIV. (A, left) Heat map of WT Lrp occupancy (same as Fig. 3A). (Right) Heat map of L136R Lrp occupancy. The *y* axis (right) contains all 517 significant L136R Lrp binding sites under at least one L136R Lrp condition, the *x* axis shows the average from both biological replicates for each lineage replicate in each medium (red, Min; blue, LIV) and each time point (white, mid-log phase; gray, stationary phase), and the color at each location represents the *lrp*::*scar*-subtracted Lrp occupancy. Both the *x* and *y* axes are clustered by Euclidean distance (taking the average distances between cluster members). (B) Pairwise Spearman correlations of the WT and L136R heat maps from panel A. (C) Transcription start site (TSS) pileup plots, where each row represents an Lrp-regulated TSS and each column is centered on the annotated TSS, showing the L136R Lrp occupancy within ±1 kb of that TSS (color), with transcription moving to the right. For each of the four conditions (columns), the L136R Lrp occupancy (color) shown represents the mean from both biological replicates of both lineage replicates. (D) Heat map of changes in RNAP occupancy for each L136R Lrp condition shown at the TUs corresponding to the TSSs in panel C. (E, left) Lrp occupancy at mid-log phase in Min (top) and LIV (bottom) at the *aroG* region. (Right) Changes in RNAP occupancy for the *aroG* TU for each of the three Lrp variants in Min and LIV at mid-log phase. Error bars represent the standard errors of the means. +, IHW *q* value of <0.1; *, IHW *q* value of <0.05; **, IHW *q* value of <0.01; ***, IHW *q* value of <0.001. (F) Overall distribution of Lrp occupancy at peaks in the indicated genotypes. The violin plots display the overall distribution of Lrp occupancy (green, WT; red, L136R) at the peaks called under each condition, whereas the inner box plot shows the 25th, 50th, and 75h percentiles of the data. Significance was calculated using the paired Wilcoxon signed-rank test; *** indicates a *P* value of <0.001. (G) Same as panel E except at the *pepD-gpt* locus.

The clustering of similar Lrp occupancy patterns in Fig. 2B revealed that L136R in LIV behaved almost identically to WT Lrp in Min (Groups III and IV), suggesting that L136R is mostly insensitive to LIV. We further characterized the L136R octamer-only mutant by generating a heat map of L136R occupancy at all 517 regions for which a significant L136R peak was called under at least one condition (Fig. 4A, right) and compared these findings with the occupancy profile of WT Lrp (Fig. 4A, left). Consistent with our findings from Fig. 2B, the L136R mutant binds to DNA with similar intensities in Min and LIV, unlike WT Lrp, as quantified by pairwise Spearman correlations of Lrp occupancy profiles between conditions in Fig. 4B. To investigate whether this leucine-insensitive effect remains true for L136R-mediated gene regulation, we plotted L136R occupancy at all 156 TSSs overlapped by an L136R Lrp peak (Fig. 4C), along with the changes in RNAP occupancy within the associated TU (Fig. 4D). Both the TSS pileup plots and the changes in RNAP occupancy were similar when comparing Min-Log with LIV-Log, with L136R occupancy decreasing only slightly in LIV-Log relative to Min-Log, demonstrating that L136R is mostly insensitive to leucine with respect to both DNA binding and gene regulation. It is also notable that unlike the case with the wild-type Lrp data in Fig. 3C, in Fig. 4D, we observe that the profile of RNAP occupancy at Lrp-associated promoters is strongly correlated under the Min-Log versus the LIV-Log conditions for the L136R mutant (Fig. 4D).

Two interesting, yet related, trends arise in the L136R mutant: in LIV, L136R Lrp behaves almost identically to the WT in Min, but in Min, L136R Lrp binds more strongly to canonical Lrp sites than WT Lrp. Figure 4E demonstrates that L136R Lrp in LIV behaves like WT Lrp in Min at the *aroG* region. Here, L136R Lrp occupancy in LIV (Fig. 4E, bottom) very closely mimics WT Lrp occupancy in Min (top). Furthermore, these similar Lrp occupancy profiles result in similar effects on gene expression: L136R in LIV activates the expression of *aroG* to an extent similar to that of WT Lrp in Min (Fig. 4E). In contrast, WT Lrp and D114E Lrp no longer bind to the *aroG* TSS in LIV and also no longer activate the expression of *aroG* in LIV (Fig. 4E). Intriguingly, in Min, L136R Lrp also binds more strongly to DNA than WT Lrp (Fig. 4F). The *pepD-gpt* region (Fig. 4G) provides an illustrative example, with L136R Lrp showing a stronger occupancy signal at the *pepD-gpt* region in Min than WT Lrp (top). While the increased occupancy of L136R at the *pepD-gpt* promoters did not have a significant differential effect on *pepD* expression in Min, it caused a significant increase in *gpt* repression relative to WT Lrp, suggesting that this increased binding to DNA is functional and leads to changes in Lrp-mediated gene regulation. Overall, our findings show that L136R is mostly insensitive to leucine and thus can bind more strongly to its binding sites than WT Lrp, which is inhibited by leucine. It is important to note that while Lrp occupancy is quantitatively higher in L136R samples than in WT samples, there do not appear to be many novel DNA binding sites in L136R relative to WT Lrp during log-phase growth in Min (Fig. S1).

### D114E Lrp has novel binding sites in the genome and generates additional peaks near existing sites.

**FIG 5:**
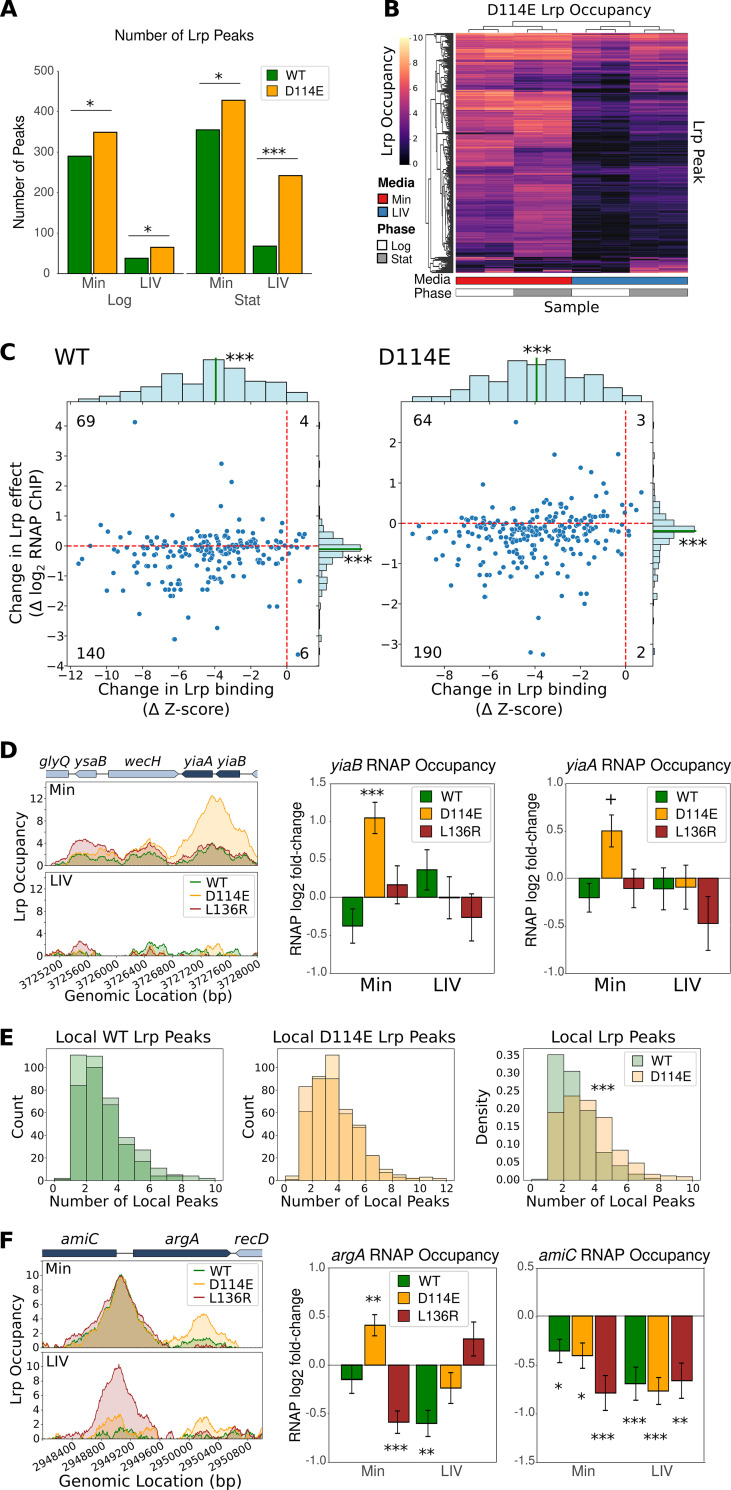
D114E has novel binding sites in the genome and generates additional peaks near existing ones. (A) Total number of peaks under each of the four conditions tested for WT Lrp and D114E Lrp. The significance of differences was assessed using the poisson.test function in R. *, *P* < 0.05; **, *P* < 0.01; ***, *P* < 0.001. (B) Heat map of D114E Lrp occupancy at all significant D114E Lrp binding sites across conditions, where the *y* axis contains all 556 significant D114E Lrp binding sites under at least one D114E Lrp condition, the *x* axis shows the averages from both biological replicates for each lineage replicate in each medium (red, Min; blue, LIV) and each time point (white, mid-log phase; gray, stationary phase), and the color at each location represents the *lrp*::*scar*-subtracted Lrp occupancy. Both the *x* and *y* axes are clustered by similarity. (C) Distributions of the changes in Lrp binding (*x* axis) versus the changes in Lrp effects on transcription (*y* axis) induced by the addition of LIV to the medium during log-phase growth, calculated for all genes where Lrp peaks overlap promoters for that genotype under at least one condition. Genotypes are indicated at the top left of each plot. Lrp-dependent expression changes are calculated by taking the change in Lrp-dependent RNAP occupancy at each promoter induced by LIV addition and multiplying this value by the sign of the occupancy in Min (thus, a positive score means that LIV strengthens the effect of Lrp, and a negative score means that LIV reduces the effect of Lrp, relative to the Min condition). Marginal distributions have their medians shown with a green line, with asterisks indicating significant differences from zero in the center of the distribution (***, *P* < 0.001 by a one-sample Wilcoxon signed-rank test). Numbers in each quadrant indicate the counts of promoters appearing in that quadrant. (D, left) Lrp occupancy at mid-log phase in Min (top) and LIV (bottom) at the *yiaB-yiaA* region. (Middle and right) Changes in RNAP occupancy for the *yiaB* TU (middle) and the *yiaA* TU (right) for each of the three Lrp variants in Min and LIV at mid-log phase. (E, left and middle) Histograms of local peak counts for both WT lineage replicates (left) and D114E lineage replicates (middle). See Text S3 in the supplemental material for details. (Right) Histogram of the density of local peak counts for the WT versus the D114E mutant, which shows a significant difference in mean counts (*P* < 0.001 by a permutation test). (F) Same as panel D except at the *amiC-argA* region.

In contrast to L136R, the D114E octamer-only Lrp mutant binds to many novel sites in the genome relative to WT Lrp, in addition to canonical Lrp sites (Fig. 2B, Classes 2, 3, and 7, and Fig. 5A). A heat map of Lrp occupancy for D114E samples at all 556 regions bound by D114E under at least one condition (compared to only 440 sites for WT Lrp) reveals that exogenous leucine strongly reduces D114E occupancy at its target sites relative to Min, suggesting that unlike the L136R octamer-only mutant, the D114E octamer-only mutant is responsive to leucine (Fig. 5B). Similar to WT Lrp, D114E Lrp occupancy at the 160 TSSs overlapped by a D114E peak was reduced in LIV-Log relative to Min-Log, and this reduction in Lrp binding was often accompanied by a reduction in the degree to which RNAP occupancy at the associated TU changed (Fig. 5C). In general, D114E appears to bind novel sites on the genome in Min and also generates novel secondary peaks in proximity to canonical WT Lrp binding sites in Min, both of which are typically disrupted in LIV.

Across all conditions, D114E had 208 unique binding sites relative to WT Lrp, but only 35 of these novel peaks (16.8%) overlapped an annotated TSS. An example of one such novel D114E Lrp peak is shown in Fig. 5D in Min at the *yiaB-yiaA* region. Unlike WT Lrp and L136R Lrp, D114E Lrp forms a strong peak at the *yiaB* and *yiaA* promoters, which is also accompanied by the strong activation of both *yiaB* and *yiaA* in Min, while WT Lrp and L136R Lrp do not significantly alter the expression of these genes (Fig. 5D).

We also observed that D114E Lrp often formed novel local secondary peaks in addition to binding to the canonical WT Lrp sites. We quantified these peaks by calculating the distribution of the number of nearby Lrp peaks around each defined Lrp peak (using a distance threshold of 2 kb to define locality) and found that the occupancy of WT Lrp had an average of 2.2 local peaks, whereas D114E Lrp showed an average of 3.1 local peaks (Fig. 5E). For example, Fig. 5F shows that D114E forms the canonical Lrp peak at the *argA* promoter (along with the WT and L136R) in Min, but also forms an additional peak within the *argA* ORF, where neither WT nor L136R Lrp is bound. Intriguingly, this additional D114E peak at *argA* is associated with significant Lrp-mediated activation of *argA*, whereas both the WT and L136R, which lack this additional peak, repress *argA* in Min (Fig. 5F). While the exact molecular mechanism giving rise to these extra binding sites in D114E Lrp strains is unclear, we hypothesize that the multiple local peaks represent higher-order (larger-than-octamer) Lrp oligomers that can then bridge multiple sites on the genome over kilobase length scales. Thus, while D114E was originally characterized as an octamer-only Lrp mutant, the D114E mutation actually most likely stabilizes higher-order Lrp oligomers in Min relative to WT Lrp, allowing it to occupy these additional sites next to canonical Lrp sites.

### Lrp forms higher-order oligomers and bridges DNA by binding to one or more nearby regions.

**FIG 6:**
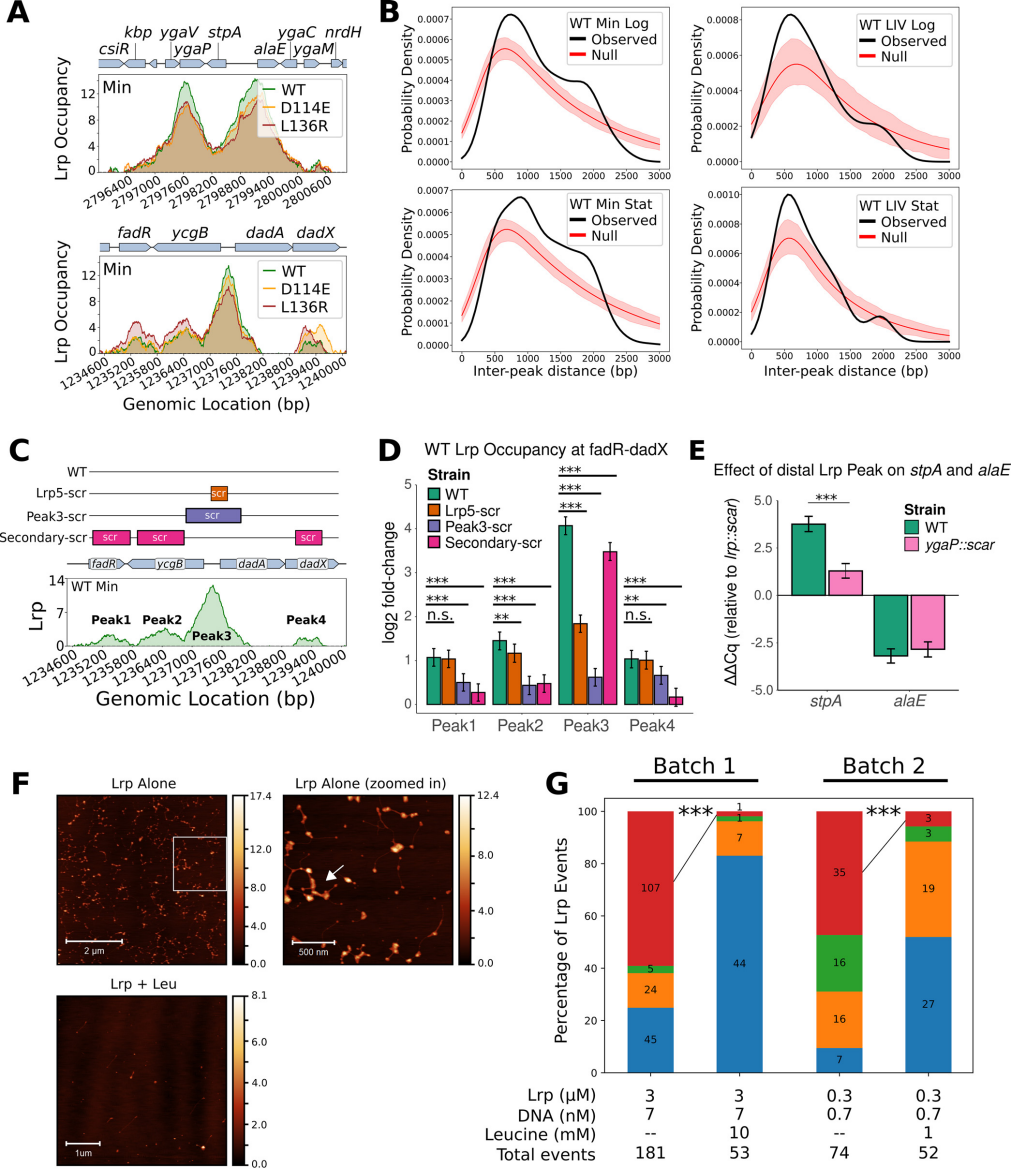
Lrp bridges the DNA locally by binding to one or more nearby regions. (A) Examples of double Lrp peaks (top) at the *stpA-alaE* region and secondary Lrp peaks (bottom) at the *fadR-ycgB-dadAX* region. In both cases, the Lrp occupancy is plotted at mid-log phase in Min. (B) Distributions of interpeak distances observed for Lrp binding sites across the indicated conditions. In each case, the actual distribution is shown in black (observed), and a simulated null distribution is shown in red, obtained through 200 random shufflings of the peak locations within each considered region (see “Calculation of the distribution of the number of local Lrp peaks” in Text S3 in the supplemental material for details). For the null distribution, the mean across samples is shown as a line, and the 95% confidence interval is shown as the shaded area. Densities are smoothed using the gaussian_kde function in scipy with default settings. (C, top) Diagram of strain mutations at the *fadR-ycgB-dadAX* locus. All constructs have an FLP recombination target (FRT)-flanked *kanR* marker immediately downstream of *dadX*. Lrp5-scrambled (Lrp5-scr) scrambles the 17-bp Lrp5 binding site within Peak 3 (19). Peak3-scrambled was carefully constructed to keep the *ycgB* and *dadAX* promoter elements and ribosome binding sites intact. All Peak3-scrambled and Secondary-scrambled sequences that overlapped an open reading frame were synonymous mutations such that the amino acid sequences remained intact but the DNA sequences varied to prevent or reduce Lrp binding. (Bottom) WT Lrp occupancy in minimal medium at mid-log phase at the *fadR-ycgB-dadAX* region, with all four peaks labeled accordingly. (D) Lrp ChIP-qPCR showing the occupancy at each of the four WT Lrp binding sites within the *fadR-ycgB-dadAX* region in the presence of the sequence variations shown in panel B. Bars shown are the means from a Bayesian model of the log_2_ fold changes of Lrp ChIP-qPCR at Peaks 1 to 4 relative to the input, compared to the average from two control regions where Lrp is known not to bind; error bars show 95% credible intervals for each case. Asterisks indicate significance calling based on the log_10_ Bayes factor (*, significance of 0.5 to 1; **, significance of 1 to 2; ***, significance of >2 [according to interpretive cutoffs reported previously {27}]). n.s., not significant. (E) Effects of Lrp binding-site mutations at the *stpA-alaE* locus on the respective transcript levels. Shown are the data from the Bayesian analysis of ΔΔ*C_q_* values from qRT-PCR of WT *ygaP* and *ygaP*::*scar* strains at *stpA* and *alaE* in Min at mid-log phase, comparing WT Lrp to the *lrp*::*scar* mutant. Error bars and significance values are the same as those in panel C. (F) AFM images of purified WT Lrp with the *stpA-alaE* DNA fragment (top left), a zoomed-in version of the region within the white box (top right), and WT Lrp (3 μM) incubated with the same DNA fragment (7 nM) in the presence of l-leucine (10 mM) (bottom). Color bars represent the height of the sample relative to the surface of the mica. (G) Relative proportions (bar graphs) and raw event counts (numbers on bars) for all observed Lrp events in the indicated samples. Note that “Batch 1” and “Batch 2” refer to two separately cross-linked batches of Lrp-DNA interactions. Asterisks indicate the results of significance tests (using the prop.test function in R) for the proportion of events falling into the “intermolecular bridging” category in the paired samples with versus without leucine, as indicated. ***, *P* < 0.001.

While D114E exhibits a propensity for forming secondary peaks within proximity to primary Lrp peaks, WT Lrp also forms both double peaks and secondary peaks (Fig. 6A) in Min but only at a subset of sites (Fig. 5E). By examining the distributions of interpeak distances across conditions, we found that in addition to a general propensity for Lrp binding sites to cluster, the interpeak distance distribution shows a substantial enrichment of distances in the 1.5- to 2.0-kb range when cells are grown in minimal medium, which is lost under the LIV condition (Fig. 6B), possibly reflecting a characteristic distance for longer-range Lrp-mediated interactions in Min. One particularly striking example is the *fadR-ycgB-dadAX* region, which contains a strong primary Lrp peak (Peak 3) at the divergent *ycgB* and *dadAX* promoters and three secondary peaks (two upstream and one downstream) in Min (Fig. 6A, bottom), separated by 1,200 to 1,800 bp. We chose to study the *fadR-ycgB-dadAX* region in order to understand what role, if any, the observed local secondary peaks play in Lrp binding within the primary Lrp peak and vice versa. If the Lrp peaks show correlated changes in binding across perturbations of different sites, this would support our hypothesis that Lrp (and, more so, D114E) forms higher-order oligomers along the DNA, especially in Min, where binding to DNA is generally stronger. We thus constructed four strains (WT, Lrp5-scrambled, Peak3-scrambled, and all secondary peaks scrambled, as shown in Fig. 6C, top [note that the amino acid sequences encoded by annotated open reading frames and the core promoter regions were preserved in all of them]) (see Text S3 for details) with perturbations to different portions of the *fadR-ycgB-dadAX* region and performed Lrp chromatin immunoprecipitation-quantitative PCR (ChIP-qPCR) on each strain to measure changes in Lrp occupancy in Min-Log relative to two control regions (in the *cysG* and *mdoG* open reading frames). Within the primary peak (Peak 3), there are 11 previously characterized Lrp dimer binding sites. Among them, the 17-bp Lrp5 binding site is thought to play a particularly important role; it is essential for the derepression of *dadAX* in LIV but is typically bound by Lrp in both Min and LIV (19).

We observed that scrambling just the Lrp5 site in the *ycgB-dadA* intergenic region significantly reduced (but did not eliminate) Lrp binding to Peak 3 while not substantially affecting Lrp binding to the secondary peaks (Fig. 6D). Furthermore, scrambling the Peak 3 sequence almost completely abrogated Lrp binding to Peak 3 (as expected) but also significantly reduced Lrp binding at the secondary peaks, suggesting that Lrp accumulation at the main peak in the divergent *ycgB-dadAX* regulatory region is required for secondary Lrp peak formation. Interestingly, scrambling all of the secondary peaks also significantly reduced Lrp binding to the primary peak (Fig. 6D; see also Table S4), although with a smaller effect in magnitude than that of scrambling the Lrp5 site, and also altered the transcription of both *ycgB* and *dadX* (Fig. S2). Since perturbing Lrp accumulation at one peak affects Lrp binding at the others nearby, it is likely that Lrp at the primary and secondary Lrp peaks forms a higher-order Lrp oligomer that bridges these DNA sites together. Furthermore, the fact that the scrambling of Lrp5 was sufficient to greatly reduce the occupancy at the primary Lrp peak suggests that the Lrp5 site (17 bp) serves as the seed sequence that nucleates Lrp binding to the primary peak (Peak 3 [~1 kb]). These results also strengthen our inference that D114E, which forms novel secondary peaks near many canonical Lrp binding regions, likely stabilizes the higher-order Lrp oligomerization state that bridges DNA in Min.

10.1128/mbio.02690-22.5 FIG S2 Effects of Lrp binding-site perturbations on the expression of genes in the *fadR-ycgB*-*dadAX* region. Shown are the results of a Bayesian analysis of ΔΔ*C_q_* values from qRT-PCR experiments comparing the expression levels of the indicated genes to those of a set of reference transcripts, of the indicated genotype (Strain), relative to WT cells. The sign convention is such that positive values indicate higher expression levels of the target gene for the indicated genotype than for the WT. It is notable that the perturbation of the secondary sites (pink bars) is sufficient to substantially alter the expression of both *ycgB* and *dadX*, consistent with our inference of interactions between the multiple Lrp binding sites at this locus being important for the regulatory output, even though the effects on binding at Peak 3 are small in magnitude. We also note that the effects on the gene expression of the secondary scramble cannot arise solely due to the modest decrease in binding at Peak 3 because in that case, *dadA* expression would also be expected to decrease (as in the case of the *lrp*::*scar* mutant). For the sake of completeness, we also include effects on transcript levels arising from the Lrp5-scr and Peak3-scr perturbations, but note that these cases should be interpreted with extreme caution because they involve changes to the sequence of the *ycgB-dadA* intergenic region. Download FIG S2, TIF file, 0.2 MB. Copyright © 2023 Ziegler and Freddolino. 2023 Ziegler and Freddolino. https://creativecommons.org/licenses/by/4.0/ This content is distributed under the terms of the Creative Commons Attribution 4.0 International license.

10.1128/mbio.02690-22.10 TABLE S4 Statistical analysis of ChIP-qPCR occupancy data. Occupancy data from Fig. 6D were analyzed using a Bayesian model as described in Text S3 in the supplemental material. “value” indicates the posterior mean, “ci.lo” and “ci.hi” are the bounds of a 95% credible interval, and “sig.vs.wt” is the posterior probability of a difference (relative to the WT strain) in the indicated direction. Download Table S4, XLSX file, 0.01 MB. Copyright © 2023 Ziegler and Freddolino. 2023 Ziegler and Freddolino. https://creativecommons.org/licenses/by/4.0/ This content is distributed under the terms of the Creative Commons Attribution 4.0 International license.

To further assess whether double or secondary Lrp peaks indeed indicate Lrp bridging DNA and have potential regulatory consequences, we next tested the effect of removing the upstream Lrp peak (which is contained within the *ygaP* ORF) at the *stpA-alaE* region (Fig. 6A, top) on the expression of the associated genes. While the removal of the upstream Lrp peak had only very minor effects on *alaE* expression, it caused a >5-fold reduction in Lrp-mediated *stpA* activation (Fig. 6E), even though the perturbed Lrp site is located over 1 kb away from the Lrp-occupied *stpA* promoter. The long-range regulatory effect observed provides further evidence that the double Lrp peak reflects a structure that acts to bridge DNA and, thus, mediate gene expression in Min.

As a direct test for Lrp-mediated DNA bridging *in vitro*, we purified native, untagged Lrp along with the *stpA-alaE* DNA fragment and performed atomic force microscopy (AFM) experiments in the absence or presence of l-leucine. As was expected based on our *in vivo* experiments, Lrp bridges DNA, but the addition of l-leucine abrogates Lrp-mediated DNA bridging (Fig. 6F and G).

## DISCUSSION

E. coli Lrp has long been hypothesized to exist in an equilibrium between hexadecamers and octamers, with exogenous leucine strongly shifting the equilibrium toward the octameric state. However, this oligomerization-based model was insufficient to mechanistically explain how Lrp utilizes six different modes of regulation of its target genes where Lrp activates or represses and leucine has no effect, augments Lrp activity, or inhibits Lrp. Through the construction of strains harboring *lrp* at a safe genetic locus in a background with fully intact branched-chain amino acid metabolism, we were able to reproduce previous findings while also providing insight into the mechanism of Lrp regulation of its target promoters. We show that the role of exogenous leucine is to inhibit WT Lrp binding to DNA and demonstrate with both our data set and a previously published data set that WT Lrp utilizes only two modes of gene regulation at its target promoters: Lrp activates or represses transcription, while exogenous leucine inhibits Lrp activity by reducing its ability to bind DNA. The other four modes of Lrp regulation at target promoters reported in the literature likely arose from indirect effects or specialized interactions also involving another regulator at a few loci.

By coupling small-scale experiments with high-throughput ChIP-seq on WT Lrp and three oligomerization mutants under various conditions, we were also able to connect the effects of leucine, the oligomerization state, and DNA binding. Dimer-only Lrp (ΔC11) was effectively unable to bind DNA despite being expressed at only slightly lower levels than WT Lrp at mid-log phase, corroborating previous *in vitro* gel shift results (12). Previous studies of D114E and L136R suggested that both mutations locked Lrp in an octamer-only state, and analyses of regulation at the *ilvIH* promoter (where the mutants were originally identified) suggested that both mutants also had the same regulatory function (13, 15). Despite using different techniques, our experiments replicated these findings at *ilvIH* in Min and LIV while also demonstrating that these two octamer-only mutants actually behave quite differently at many genomic sites, with the common behavior at *ilvIH* in fact representing a case of concordance that is not universal. Our results indicate that L136R is truly a leucine-insensitive Lrp mutant. In contrast, D114E responds almost as strongly to exogenous leucine as WT Lrp but overall binds to more sites in the genome and globally forms secondary peaks near canonical Lrp sites. These findings suggest that D114E is indeed an oligomerization mutant but likely not octamer-only; instead, we propose that D114E favors the formation of even higher-order oligomers (e.g., hexadecamers) in minimal media that bridge DNA. Our ChIP-qPCR and quantitative reverse transcriptase PCR (qRT-PCR) experiments on strains with mutated sequences at the locations of double and secondary Lrp peaks demonstrate that the Lrp complexes binding nearby peaks likely interact with each other. Indeed, our AFM experiments directly demonstrate that Lrp bridges DNA but that Lrp-mediated DNA bridging is abrogated in the presence of leucine. The bridging behavior and associated effects on Lrp-mediated gene regulation that we observed at the *stpA-alaE* promoter are likely characteristic of similar mechanisms at play in other regions with multiple Lrp binding peaks.

E. coli Lrp belongs to an ancient and highly conserved class of proteins called feast-famine response proteins (FFRPs), with homologs in almost all sequenced archaea and nearly half of all sequenced bacteria (3). Across all FFRPs, four small-molecule effector binding sites have been identified (20). Two such sites (type I and type III) are found in E. coli Lrp, where the type I site is the highly conserved high-affinity effector binding site and type III is less conserved and serves as a low-affinity effector binding site (6). Previous studies have reported that residue D114 in Lrp is part of the low-affinity site and contributes to higher-order Lrp oligomerization (13) (see Fig. S3A in the supplemental material). This is consistent with our findings that the D114E mutation stabilizes higher-order oligomers (e.g., hexadecamers). Conversely, the Lrp L136R mutation likely disrupts the type I (high-affinity) effector binding site (20–22), which is consistent with our findings that L136R Lrp is mostly leucine insensitive (Fig. S3B). Collectively, our findings demonstrate that neither D114E nor L136R is an octamer-only mutant, but rather, their unique phenotypes are connected to the roles of high- and low-affinity effector binding sites on Lrp in altering DNA binding (high-affinity site, L136R) and oligomerization (low-affinity site, D114E). We expect that future experiments more directly probing the oligomerization state of Lrp at different genomic loci and directly identifying any long-range contacts formed by Lrp oligomers will provide further mechanistic insight into gene regulation by Lrp and other FFRPs. Contrasting the behaviors of the D114E and L136R mutants may also prove useful in the future for identifying the likely binding sites of newly identified Lrp effectors.

10.1128/mbio.02690-22.6 FIG S3 Location of residues D114 and L136 on the Lrp octamer structure. (A) Crystal structure of octameric Lrp (PDB accession number 2GQQ) in orange, with D114 residues highlighted in cyan. The image on the right is rotated 90° along the vertical axis relative to the image on the left. (B) Same as panel A except that the L136 residues are highlighted in cyan. Download FIG S3, TIF file, 2.5 MB. Copyright © 2023 Ziegler and Freddolino. 2023 Ziegler and Freddolino. https://creativecommons.org/licenses/by/4.0/ This content is distributed under the terms of the Creative Commons Attribution 4.0 International license.

## MATERIALS AND METHODS

### Experimental methods.

All experimental procedures are described in detail in Text S3 in the supplemental material (24–26, 30–44). In brief, we used previously described procedures for Lrp ChIP-seq (5), RNA polymerase ChIP-seq (23, 24), and Miller assays (24, 25). Western blotting was performed using standard methods, and blots were analyzed using ImageJ software. ChIP-qPCR entailed Lrp ChIP as described above, followed by qPCR using Bio-Rad iTaq universal SYBR green supermix on a Bio-Rad CFX Opus 384 instrument according to the manufacturer’s instructions (see Text S3 for details). A list of all primers and oligonucleotides used over the course of the study can be found in Table S3.

10.1128/mbio.02690-22.9 TABLE S3 Primers and oligonucleotides used in the present study. Download Table S3, XLSX file, 0.01 MB. Copyright © 2023 Ziegler and Freddolino. 2023 Ziegler and Freddolino. https://creativecommons.org/licenses/by/4.0/ This content is distributed under the terms of the Creative Commons Attribution 4.0 International license.

### High-throughput sequencing data analysis.

Sequencing data were analyzed using an updated version of the processing pipeline described previously (24) to obtain occupancy scores. For Lrp ChIP-seq, the signal from *lrp* knockout cells under the same condition was subtracted from the occupancy scores prior to analysis. For RNA polymerase ChIP-seq, we summarized the counts of aligned reads at the level of transcription units, followed by differential expression calling using deseq2 (26). Further details are given in Text S3.

### Data availability.

All high-throughput sequencing data related to this study are available at the GEO under accession number GSE198120. The full set of AFM images used in our analysis is available at https://doi.org/10.6084/m9.figshare.21836319.
