# Supplementary material for: Escherichia coli Leucine-Responsive Regulatory Protein Bridges DNA In Vivo and Tunably Dissociates in the Presence of Exogenous Leucine
Source: mBio. 2023 Feb 14;14(2):e02690-22. doi: 10.1128/mbio.02690-22 (PMC10127797; doi:10.1128/mbio.02690-22)
Supplement: TEXT S1 [file mbio.02690-22-s0002.pdf]

Text S1. Construction of a faithful genetic background for analyzing Lrp function

Many previous studies of *E. coli* Lrp utilized strain backgrounds with mutations in one or more of the three acetohydroxyacid synthase (AHAS) genes: *ilvBN*, *ilvGM*, or *ilvIH*, which are involved in the regulation and biosynthesis of branched-chain amino acids [28]; for example, the commonly used K12 strain MG1655 has a frameshift mutation in *ilvG*, whereas *ilvB* and *ilvIH* were mutated in some of the original lrp studies. Because Lrp responds strongly to the presence of L-leucine and regulates branched-chain amino acid transport and biosynthesis, we opted to use the RL3000 lab strain of *E. coli* for all of our experiments, which is a derivative of MG1655 harboring a functional *ilvG* gene, and therefore containing all three fully functional AHAS isozymes [29].

To preserve Lrp regulation and copy number, we chose to integrate all Lrp mutants directly into the genome; however, the native *lrp* locus was difficult to modify without disrupting several LexA sites downstream of *lrp* or altering *ftsK* expression, which is an essential gene involved in cell division that is located immediately downstream of *lrp*. To circumvent these issues, we instead deleted the entire open reading frame of *lrp* at its native locus, leaving behind an FRT scar (*lrp::scar*), and integrated a cassette containing the native *lrp* promoter and WT *lrp* gene flanked by strong bidirectional terminators into a locus immediately downstream of the native *thyA* gene (Fig. 1A). We chose the *thyA* locus because *thyA* encodes Thymidylate Synthase, which can be used as a Lrp cassette-linked selectable marker on media lacking thymidine, and because the *thyA* and *lrp* loci are roughly equidistant from the origin and therefore have similar DNA copy numbers. To investigate the effect of oligomerization Lrp mutants on gene expression, we also integrated the “dimer-only” ΔC11 and the “octamer-only” D114E and L136R *lrp* genes into the thyA locus using the same approach (a full list of strains used in our study is given in Table S1).
