# Supplementary material for: Escherichia coli Leucine-Responsive Regulatory Protein Bridges DNA In Vivo and Tunably Dissociates in the Presence of Exogenous Leucine
Source: mBio. 2023 Feb 14;14(2):e02690-22. doi: 10.1128/mbio.02690-22 (PMC10127797; doi:10.1128/mbio.02690-22)
Supplement: TEXT S3 [file mbio.02690-22-s0001.pdf]

Supplementary Methods

Bacterial Strains and Culturing:

The parental strain, RL3000 lrp::scar, was generated via P1vir transduction of *lrp::KanR* from the Keio collection [30] strain JW0872 into RL3000 followed by electroporation of pCP20 to flip out the FRT-flanked KanR marker. From here, *thyA::KanR* was transduced from the Keio collection strain JW2795 via P1vir transduction [31] and selected on LB plates supplemented with kanamycin and 20 µg/mL thymidine, followed by electroporation of pKD46 [32]. Plasmids containing *lrp* (with its native promoter) flanked by strong bidirectional terminators and linked to *thyA* were generated for WT *lrp* and each of the *lrp* mutants or *thyA* only for the Δ*lrp* strains. Final strains were constructed via lambda red recombination of the *thyA*-*lrp* cassette into the pKD46-bearing lrp::scar, thyA::KanR strains, selected on LB lacking thymidine, and confirmed via colony PCR and Sanger sequencing. RL3000, *lacZ::CAT,* p_{oppA}-*lacZ,* and RL3000*, lacZ::CAT,* p_{alaE}-*lacZ* strains used for the Miller Assays were generated by first using lambda red recombination to replace native *lacZ* with *CAT* in the RL3000 lrp::scar parent, followed by introduction of thyA::*kanR* via P1vir transduction. From here, a cassette containing *lacZ* linked to loxP-flanked *specR* was recombined into the genome at either native p_{oppA} or p_{alaE}, such that the cassette was integrated between the promoter and translation start codon of *oppA* or *alaE*. Finally, *specR* was flipped out via induction of Cre recombinase from an arabinose-inducible plasmid, and the appropriate *lrp* mutant was integrated into the *thyA* locus using P1vir from the *thyA*-*lrp* strains from above. All cloning was performed in standard LB media supplemented with appropriate antibiotics. All strains used in experimental measurements (with the exception of the parental RL3000) were constructed in duplicate to ensure the robustness of our results; we refer to the independent but nominally isogenic variants of each genotype as "lineage replicates".

For physiological experiments such as ChIP-seq, single colonies were inoculated into 0.04% glycerol-ammonia M9 minimal media and grown overnight at 37°C. The next day, these overnight cultures were diluted 1:200 into 0.4%-glycerol-ammonia M9 media (Min) or 0.4%-glycerol-ammonia M9 supplemented with 0.2% each of L-leucine, L-valine, and L-isoleucine (LIV). Log-phase samples were collected at OD_{600}=0.2 and stationary-phase samples were collected approximately 12 hours after the log-phase time-point. Each "biological replicate" for such experiments refers to an experiment performed on a separate day arising from a separate colony grown as described above.

Miller Assays:

Single colonies from LB plates were inoculated into 3mL 0.04%-glycerol-ammonia M9 minimal media and grown at 37°C overnight. The next day, 5µL cells were added to 1mL of Min or LIV minimal media (see above) in 2mL wells of a deep 96-well plate and grown at 37°C with shaking until approximately the desired OD_{600} was reached (around 0.2). At this point, 80µL cells were taken and added to a clear 96-well plate and the OD_{600} of each well was measured on a plate reader. 120µL of the **β**-Galactosidase master mix (80µL Z-Buffer with 2.7µL/mL **β**-mercaptoethanol, 30µL Z buffer with 4mg/mL ortho-nitrophenol-galactoside (ONPG), 8µL PopCulture detergent (EMD Millipore Corp., Billerica, MA), and 2µL10mg/mL lysozyme in water, where Z-Buffer consists of 60mM Na_2HPO_4, 40mM NaH_2PO_4, 10mM KCl, and 1mM $MgSO_4$) was then carefully mixed into each well with cells and the OD_{550} and OD_{420} were monitored every two minutes for one hour in a plate reader

with shaking between readings. RL3000 in the presence of 1mM IPTG was used as a positive control, whereas RL3000, *lacZ::CAT* was used as the negative control in these experiments. This protocol was adapted from [25] and [24].

Western Blots:

Single colonies from each strain were inoculated into 5mL LB and grown overnight at 37℃, back-diluted 1:200 into 3mL 0.04% Glycerol-Ammonia M9 media and grown again overnight at 37℃. The OD_{600} of the resulting overnight cultures was measured and the appropriate amount of cells were added each into 5mL 0.4% Glycerol-Ammonia M9 media (Min) and 5mL 0.4% Glycerol-Ammonia M9 media supplemented with 0.2% each of L-leucine, L-isoleucine, and L-valine (LIV) such that the starting OD_{600} in these cultures was 0.003. Cultures were grown at 37℃ with shaking until mid-log phase (OD_{600} = 0.2), at which point 2mL was spun down and cell pellets were flash-frozen at -20℃. Approximately 12 hours after the mid-log time-point was taken, the OD_{600} was again measured, 1mL of these stationary phase samples were spun down, and cell pellets were flash-frozen at -20℃.

Cell pellets were resuspended in 1x Laemmli Loading Buffer (5µL 4x Laemmli Buffer: 1µL 1M DTT: 14µL 1xPBS) such that the concentration of cells was OD_{600}=10/mL. Solutions of 0.5µM, 1µM, and 5µM solutions of purified native Lrp in 1x Laemmli Loading Buffer in order to calculate the amounts of Lrp in each sample and across blots. Samples were denatured at 99℃ for 10min. 7µL of Bio-rad Precision-Plus Protein All-Blue Standards and 10µL each sample were then loaded into the wells of 15well 4-20% Bio-rad Mini-PROTEAN TGX Stain-Free Gels and electrophoresed at 175V for 45min at room temperature in Tris-Glycine-SDS Running Buffer. Gels were then rinsed in milliQ water and total protein was imaged with the 5min Stain-Free gel protocol on the Bio-Rad ChemiDoc MP Imaging System. Proteins were transferred onto Bio-rad Immuno-Blot PVDF Membranes (pre-activated in 100% methanol) via the wet transfer technique in Tris-Glycine-Methanol Transfer Buffer at 60V for one hour at 4℃. PVDF membranes were briefly rinsed in milliQ water and then incubated with 30mL 3% non-fat milk in TBST (Tris-Buffered Saline with Tween20) with gentle shaking at room temperature for 30 minutes. Excess milk was removed from the membrane and 10mL 1:4,000 monoclonal Lrp antibody in 3% non-fat milk in TBST was added to each membrane, incubating for 2 hours at room temperature. Primary antibody solution was removed and 10mL 1:5,000 HRP-goat, anti-mouse antibody in 3% non-fat milk in TBST was added to the membrane and allowed to incubate overnight at 4℃. The next day, PVDF membranes were washed 3x with TBST, allowing the membranes to gently shake at room temperature for 10min between washes. After washing, the PVDF membranes were rinsed briefly with milliQ water and 1mL of MilliPore Immobilon Western Chemiluminescent HRP Substrate was added to the top of the membrane for 1 minute prior to imaging. Images were taken with a two-second exposure in the Chemiluminescence protocol on the Bio-Rad ChemiDoc MP Imaging System .

Raw image (tiff) files from the Lrp western blot images were loaded into ImageJ software and rectangles of the same size were placed over each Lrp band across the blot. Background signal was subtracted and the total pixel density was calculated for each band. Total protein signal was measured in a similar manner by loading the total protein gel image tiff files into ImageJ and, instead of placing a rectangle around a specific band, a rectangle was placed around each lane of the gel. After background subtraction, the total pixel density from each lane was calculated. To normalize Lrp levels and generate

relative Lrp abundances on the Western Blots, the Lrp western blot band pixel density was divided by the total protein blot lane pixel density for each sample.

The relative Lrp abundances were then analyzed using a Bayesian model, in which we assumed population-level (fixed) effects for the biological condition (media/growth phase combination), sample genotype, genotype:media interaction, genotype:growth phase interaction, and genotype:condition interaction, as well as group level (random) effects for each specific gel and for the effects of the condition given the strain identity. Data were log_2-scaled and z-scored prior to model fitting, and then we report the back-transformed parameters in the original data units. The models were fitted using the R package brms [33,34] with 5000 iterations in each of four Monte Carlo chains; convergence was assessed based on the Rhat criterion plus visual inspection of the posterior predictive distributions. We used a normal(0,3) prior for the population-level effects, a normal(0,2) prior for the group-level effects, an lkj(10) prior for correlation parameters, and default priors for all other parameters.

Paired Lrp-ChIP-seq and RNAP-ChIP-seq:

Single colonies of each lineage of each strain were first grown overnight at 37°C in 0.04%Glycerol, 0.4%NH_4Cl M9 minimal media with shaking, The next day, cells were back-diluted to OD_{600}=0.003 into 250mL 0.2%Glycerol, 0.4%NH_4Cl M9 minimal media (Min) or 250mL 0.2%Glycerol, 0.4%NH_4Cl M9 minimal media supplemented with 0.2% each of L-leucine, L-isoleucine, and L-valine (LIV) and grown at 37℃ with shaking until OD_{600}=0.2 (log-phase), at which point 100µL cells were serially diluted in 1x PBS and plated on LB plates in order to calculate CFU/mL for each sample, and three separate aliquots of 30mL cells were added to 810µL 37% formaldehyde solution (final concentration = 1%), and 300µL 1M $NaPO_4$, pH=7.4 and incubated with shaking at room temperature for 15 minutes. To quench the crosslinking reactions, 4M Tris, pH=8.0 was added to a final concentration of 280mM and samples were incubated with shaking at room temperature for an additional ten minutes. Cells were spun down at 15,000 x g for 2minutes at 4℃ and cell pellets were washed twice with 50mL ice-cold TBS (50mM Tris, pH=7.5, 150mM NaCl). Final cell pellets were resuspended in 1mL TBS, transferred to 1.7mL Eppendorf tubes, and centrifuged for 3 minutes at 12,000 x g at 4℃. Upon removal of supernatant, cells were flash-frozen in a dry-ice ethanol bath and stored at -80℃. The same protocol was followed for the stationary phase time-point, which occurred 12hrs after removal of the log-phase aliquots. Note that for each sample and time-point, three cell pellets were generated.

Two of the frozen cell pellets for each log-phase sample were resuspended and combined in a total of 600µL Lysis Buffer and vortexed for 3 seconds. For stationary-phase samples, one pellet was resuspended in 1mL PBS and 100µL of this solution was added to 500µL Lysis Buffer. All samples were incubated at 37℃ for 30min and then placed on ice. Samples were kept cold and sonicated for three bursts of 10s ON, 10s OFF at 25% power. 6µL RNaseA (10mg/mL), 5.4µL 100mM $MnCl_2$, 4.5µL 100mM $CaCl_2$, and 6µL DNaseI were added to each sample and mixed gently. Samples were incubated on ice for 15min and quenched with 50µL 0.5M EDTA, pH=8.0. Cell debris was removed via centrifugation at 16,000 x g for 10min at 4℃.

Cell lysates were added to 50µL pre-washed Protein G beads (supernatant from 50µL beads was discarded and beads were washed 3x with 1mL PBS+0.1% Tween20, final beads were resuspended with wide-bore tips in 50µL PBS+0.1% Tween20) and gently rocked at room temperature for one hour

to pre-clear the lysates. Samples were then placed on a magnetic stand and the supernatant was transferred to a fresh tube. 50µL pre-cleared lysate was set aside as the Input sample and mixed with 450µL Elution Buffer. Input samples were incubated for 12-16hrs overnight at 65℃ to reverse crosslinks. The remainder of the cell lysate was split in half: 275µL for the RNAP-ChIP and 275µL for the Lrp-ChIP.

For the Lrp-ChIP, 100µL Protein G beads were washed 3x in 1mL PBS+0.1%Tween20 and resuspended in 100µL PBS+0.1%Tween20. These pre-washed beads were incubated with 10µg Lrp monoclonal antibody for 10 minutes at room temperature with gentle shaking. The 275µL Lrp-ChIP sample was then added to the bead-antibody mixture and incubated at room temperature for 1 hour with gentle rocking. The beads were then washed 3x with 1mL PBS+0.1%Tween20. 500µL Elution Buffer was added to the beads and samples were incubated at 65℃ for 25 minutes with vortexing every 5 minutes. Samples were placed on a magnet stand and supernatant was placed into a fresh tube. Crosslinks were reversed by incubating overnight (12-16hrs) at 65℃.

The 275µL of RNAP-ChIP samples were added to 275µL 2x IP Buffer (1x composition: 100 mM Tris, pH 8; 300 mM NaCl; 2% Triton X100) and incubated with 10µg RNAP-ChIP antibody overnight with gentle rocking at 4℃. The next day, 50µL Protein G beads (per sample) were placed on a magnet stand and washed once with 1mL 1x IP Buffer and resuspended in 50µL 1x IP Buffer. These pre-washed beads were then added to the lysate-antibody mixture and incubated for 2hours at 4℃ with gentle rocking. For the next wash steps, 1mL of buffer was added, tubes were mixed thoroughly by inversion, placed back on the magnet stand, and supernatant discarded in the following order: IP Wash Buffer A (100 mM Tris, pH 8; 250 mM LiCl; 2% Triton X-100; 1 mM EDTA), IP Wash Buffer B (10 mM Tris, pH 8; 500 mM NaCl; 1% Triton X-100; 0.1% sodium deoxycholate; 1 mM EDTA), IP Wash Buffer C (10 mM Tris, pH 8; 500 mM NaCl; 1% Triton X-100; 1 mM EDTA), 1x IP Buffer supplemented with 1mM EDTA, and 1x TE. Beads were then resuspended in 500µL Elution Buffer (50 mM Tris, pH 8; 10 mM EDTA; 1% SDS) and incubated at 65℃ for 25minutes with vortexing every 5 minutes. Finally, samples were placed on a magnet stand and supernatant was removed to a fresh tube. Crosslinks were reversed by incubating overnight (12-16hrs) at 65℃.

After treatment with 10µL RNaseA (10mg/mL) for 2hours at 37℃ followed by 10µL Proteinase K for 2hours at 50℃. DNA from the Input, Lrp-ChIP, and RNAP-ChIP tubes was then extracted via phenol-chloroform precipitation and concentrated via ethanol precipitation.

Preparation of DNA samples for Illumina Sequencing:

Concentrations of DNA samples were determined using dsDNA Quantifluor (Promega) following manufacturer's instructions, and samples were diluted to 5ng/µL prior to performing Illumina sequencing preps with the NEBNext Ultra II DNA Library Prep Kit, following manufacturer's instructions, with the following modification to the first bead-cleanup step: 68µL isopropanol and 174µL magnetic beads were added to each sample and then washed twice with fresh 80% ethanol as normal. All sequencing preps were performed using the Opentrons2 Liquid-handling robot up through the final PCR amplification step. Final bead cleanups were performed by hand, and DNA concentrations were again determined via dsDNA Quantifluor (Promega) following manufacturer's instructions. Samples were pooled into a 5nM library and sequenced on an Illumina NovaSeq 6000.

Processing of Illumina reads and peak calling:

Read preprocessing, alignment, and initial quantitation was performed using version 2.3.6 of the IPOD-HR [24] analysis pipeline (https://github.com/freddolino-lab/ipod), up to but not including the ChIP subtraction stage. In brief, reads are clipped to remove identifiable Illumina adapter sequences, quality trimmed from their 3' ends, and then aligned to the E. coli MG1655 genome (version U00096.3) using bowtie2. Coverages were then calculated for each sample across the genome at 5 bp intervals, and the coverages quantile normalized separately for each condition/genotype combination. After quantile normalization, the median value of each sample was set to 100 by rescaling the data, and a pseudocount of 0.25 added to each position. Log_2 ratios for each non-input sample were calculated relative to the matched input sample; values were then averaged across biological replicates. The averages were subsequently converted to robust z-scores.

For the Lrp ChIP data, to remove any contributions from nonspecific antibody background, the position-wise robust z-scores from *lrp* knockout cells under the same condition were subtracted from the robust z-scores for each sample of interest; the background-subtracted robust z-scores were then used for analysis throughout the text unless otherwise noted.

Lrp peaks were called for each sample for a range of z-score thresholds from 1-8 in intervals of 0.1. We then determined (and subsequently applied) an optimal peak z-score threshold for each sample by maximizing the Kullback-Leibler divergence between the distributions of occupancy scores for peak vs. non-peak regions of the genome (with the occupancy scores discretized into bins at unit increments from -10 to 10, with a pseudocount of 1 added to each bin). Final peak lists for each sample were generated by keeping only the peaks that occurred in both lineages of a single condition with at least a 50% reciprocal overlap. Heatmaps of Lrp occupancy at each peak were generated by plotting the average Lrp occupancy across the length of a peak.

TSS pileup plots:

Occupancies around transcription start sites were collected on 2 kb windows centered on all RegulonDB-annotated transcription start sites [35] (using RegulonDB Release 10.9, downloaded 6/29/2021), and then plotted using the clustermap function of seaborn, building on the numpy, scipy, matplotlib, and pandas libraries [36–40].

RNAP-ChIP analysis:

RNAP ChIP reads were processed as described above up through the read alignment stage, and then coverage of transcriptional units (from RegulonDB [35]) calculated using the summarizeOverlaps function of the R package GenomicAlignments [41], using mode "IntersectionStrict". Differential expression calling and fold change estimation between conditions was then performed using deseq2 [26], with multiple hypothesis testing correction using the IHW method [42].

Calculation of the distribution of the number of local Lrp peaks:

To calculate the number of sub-peaks within each called peak region, we first defined a window around each peak call that contained the full peak plus 2 kb of padding in either direction (using `bedtools slop`), and then smoothed the occupancy with a Hann filter over a 500 bp window (using the `scipy.signal.convolve` function), and flagged peaks using the scipy.signal.find_peaks function

with parameters `height=1, distance=50` (corresponding to a 250 bp range between peaks, as data points were taken at 5 bp intervals). The numbers of distinct peaks identified in each window were used to generate the distributions shown in Fig. 5E.

Lrp-ChIP-qPCR:

In order to obtain the various binding site variants shown in Fig. 6B, a plasmid containing WT fadR-ycgB-dadAX upstream of a KanR marker was generated and used as a backbone template to construct the Lrp5_scr, Peak3_scr, and Secondary_scr plasmids, where the inserts were generated using the gBlocks listed in Table S3. WT RL3000 containing the λ_{red} helper plasmid (pKD46) was electroporated with a chloramphenicol acetyltransferase (CAT) gene flanked by sequences with 20bp homology to the regions upstream of *fadR* and downstream of *dadX* respectively and grown on LB plates containing chloramphenicol. Successful fadR-ycgB-dadAX knockouts were verified by PCR and Sanger Sequencing. Two independent colonies were then grown in liquid culture and electroporated with fresh pKD46 helper plasmid in preparation to generate the final strains via λ_{red} recombineering. Two independent lineages (A and B) for each of *WT fadR-ycgB-dadAX-KanR*, *Lrp5_scr–fadR-ycgB-dadAX-KanR*, *Peak3_scr-fadR-ycgB-dadAX-KanR*, and *Secondary_scr-fadR-ycgB-dadAX-KanrR* were then generated at the native genomic *fadR-ycgB-dadAX* locus using λ_{red} recombination with a PCR product of the corresponding plasmid DNA, selecting for Kanamycin resistance. Because Peak3 is about 1kb wide and sits on the divergent *ycgB* and *dadAX* promoters, we scrambled the regions not overlapping the -35 and -10 sites and made synonymous mutations in the open reading frames (ORFs) so as to minimally disrupt the proteins synthesized from these genes. Similarly, because the secondary peaks were each about 500bp and overlapped *fadR*, *ycgB*, and *dadX* ORFs, we made synonymous mutations such that the encoded proteins would be intact but the Lrp binding sites on the DNA would be disrupted. For harvest of chromatin for ChIP-qPCR, each of the target strains was grown to mid-log phase in Min media as described in "Preparation of samples for paired Lrp-ChIP-seq and RNAP-ChIP-seq" above.

Input DNA samples were diluted 1:150 in 1xTEe such that their concentrations were in the same range as the Lrp-ChIP samples. qPCR was performed on three technical replicates of each sample with Bio-Rad iTaq Universal SYBR Green Supermix according to manufacturer's instructions and samples were run on the Bio-Rad CFX Opus 384 Real-Time PCR instrument to determine Cq values. Lrp-ChIP Cq values at each locus were offset by the mean Cq value of two control regions: *cysG* and *mdoG*. See Table S3 for primer sequences. To ensure that primer efficiencies were comparable across samples/mutants, we ran qPCR with all appropriate primer pairs on a 10-fold dilution series of the input samples (ranging from 1 to 10^{-8}) and analyzed the slopes of the resulting Cq values vs. $\log_2$ dilutions. Because all of the slopes were highly comparable (-2 +/- 0.1), we did not further normalize Cq values by primer efficiency.

The ChIP-qPCR data were analyzed using a Bayesian model in which the $\log_2$ fold changes (i.e., difference between ChIP and input Cq values, after correction for the control regions) were treated using a linear model with normally distributed residuals. We included population-level terms for the peak identity (i.e. primer pair), peak:sample interaction (the key parameter of interest), and sample:lineage interaction (accounting for variability across biological replicates), as well as group-level effects for each peak:sample:lineage combination and each peak:technical replicate combination.

Models were fit using brms [33,34] using default parameters, and fit quality assessed using the Rhat criterion and visual inspection of the posterior predictive distributions. We used normal(0,10) priors for the peak terms, normal(0,2) priors for the other population-level terms, and brms default priors for all other model terms.

RNA Extraction and qRT-PCR:

Two independent lineages (A and B) of WT-lrp, *lrp::scar*, WT-lrp *ygaP::scar* and *lrp::scar ygaP::scar* were generated independently with *lrp* at the *thyA* locus as described in Figure 1A. Each strain was grown to mid-log phase (OD_{600}=0.2) in Min media (M9 Glycerol-Ammonia) as described in "Bacteria Strains and Culturing" above. At the appropriate OD_{600}, 1mL cells were mixed with 2mL RNA Protect Bacteria Reagent (Qiagen, Hilden, Germany) and incubated for five minutes at room temperature prior to pelleting the cells via centrifugation at 10,000 x g for five minutes at 4°C. The supernatant was removed and cell pellets were flash-frozen in a dry ice-ethanol bath prior to being stored at -80°C. To extract the total RNA, cell pellets were resuspended in 100µL TE and treated with 1µL ReadyLyse lysozyme solution (Epicentre, Madison, WI) and 10µL Proteinase K (Thermo Fisher, Waltham, MA) for 10min at room temperature with vortexing every two minutes. Total RNA was purified using the Zymo RNA Clean and Concentrator-5 kit according to manufacturer's instructions and eluted with 25µL nuclease-free water. To remove DNA contamination, the RNA samples were mixed with 58µL RNase-free water, 10µL Baseline Zero 10x DNase Reaction Buffer, 2µL murine RNase inhibitor, and 5µL Baseline Zero DNase and incubated at 37°C for 30min. Remaining total RNA was then purified via the Zymo RNA Clean and Concentrator-5 kit according to manufacturer's instructions and eluted with 15µL nuclease-free water. RNA was quantified using the NanoDrop and diluted to approximately 15ng/µL in nuclease-free water.

qRT-PCR was performed on three technical replicates of each sample with Bio-Rad iTaq Universal SYBR Green Supermix and Reverse Transcriptase according to manufacturer's instructions and samples were run on a Bio-Rad CFX Opus 384 Real-Time PCR instrument to determine Cq values. To ensure RNA samples did not have DNA contamination, control reactions were performed in the absence of Reverse Transcriptase. ΔΔCq values at each locus were determined by subtracting *lrp::scar* Cq values from the Cq values of the respective WT-lrp samples, and ΔΔCq values were normalized across samples by subtracting the respective mean Cq values of two control regions: *cysG* and *mdoG*.

The qRT-PCR data were analyzed using a Bayesian model similar to that described above for ChIP-qPCR data, using the Cq values from the instrument after centering the mean of all Cqs on zero. For both of the loci considered, we used a linear model in which error terms were assumed to be normally distributed, fitted using the brms R package [33,34]. In the case of the stpA-alaE locus, we fitted a model including population-level terms for the target gene, ygaP status, and three-way interaction between target gene, ygaP status, and lrp status (WT or scar); we also included group-level terms for each actual strain (combination of ygaP status, lrp status, and lineage replicate), and for each combination of gene and technical replicate. We used a normal(0,10) prior for the gene level effects, normal(0,4) priors for all other population-level terms, and brms default priors for all other terms. The key object of inference was then the difference in Cq induced by Lrp for the gene of interest (stpA or alaA), relative to the control genes, and whether those effects differed based on ygaP status.

Our analysis at the dadAX locus was similar, and in this case included population-level terms for the target gene, the cell genotype, and the target-genotype interaction, as well as group-level effects for each biological sample (genotype - biological replicate - lineage replicate combination) and each combination of target gene and technical replicate. We used normal(0,10) priors for the gene level effects, normal(0,4) priors for all other population-level effects, and brms defaults for all other priors. In this case the key object of inference was the difference in expression of each target gene (relative to housekeeping controls) observed for each genotype. Convergence and model fits were assessed as described above for ChIP-qPCR data.

Purification of native, un-tagged E. coli Lrp:

The pColdIV expression system [43,44] was used to over-express un-tagged *lrp* in an MG1655 *lrp::scar* background strain. After optimizing expression conditions, 8L of cells were grown in TB media supplemented with ampicillin at 37°C and protein expression was induced at OD_{600}=1.5 by shifting the temperature to 16°C and simultaneously adding 1mM IPTG. Cells were then grown for 42 hours at 16°C and then pelleted by centrifugation. 61g cell pellet was gently resuspended in 500mL Lysis Buffer (TGED (20% glycerol) + 0.5M KCl, pH=7.5 and sonicated for a total of 5 minutes (5s ON, 20s OFF) on ice. Sonicated lysate was then centrifuged at 15,000 x g for two hours at 4°C to remove cellular debris. The remaining lysate was dialyzed against TGED + 50mM KCl, pH=8.0 to remove excess salt. The dialyzed sample was then loaded onto a pre-equilibrated (TED +10mM KCl, pH=8.0) 50mL Q-Sepharose ion exchange column, washed with 100mL TED + 10mM KCl, pH=8.0, and eluted with a 100mL gradient from 50mM to 500mM KCl in TED, pH=8.0 buffer. Fractions containing Lrp were pooled and concentrated down to 10mL by centrifugation in the 10kDa MWCO Amicon filter units prior to being loaded onto a pre-equilibrated (TED + 0.3M KCl, pH=7.5) size exclusion column and eluted with 120mL TED + 0.3M KCl, pH=7.5 buffer. Samples containing Lrp were again pooled and dialyzed against TED + 50mM KCl, pH=7.5 to remove excess salt prior to being loaded onto a pre-equilibrated 25mL Heparin-Sepharose column (TED + 10mM KCl, pH=7.5). The Heparin-Sepharose column was then washed with 250mL TED + 10mM KCl, pH=7.5 and sample was eluted with a 50mL gradient of 10mM to 500mM KCl in TED, pH=7.5. Fractions containing Lrp were pooled and concentrated using the Amicon 10kDa MWCO filter. Glycerol was added to a final concentration of 50% w/v and purified protein was aliquoted into eppendorf tubes prior to storage at -80°C. The final concentration after addition of glycerol was 213.3µM Lrp (monomer) (4mg/mL) in a volume of 8mL (32mg purified Lrp from an 8L culture) at greater than 97% purity (based on a Coomassie stained gel).

Atomic Force Microscopy:

Purified Lrp was incubated with purified 2,101bp *alaE* DNA using primers alaE-stpA_fwd and alaE-stpA_rev (Table S3) with or without leucine in HEPES buffer (20mM HEPES, 1mM $MgCl_2$, and 100mM KCl, pH=7.5) and incubated at 37°C for 15min; concentrations are specified in Figure 6. Samples were crosslinked with 0.2% glutaraldehyde for 10min at room temperature and quenched with Tris, pH=7.5 at a final concentration of 333mM. 10µL of sample was adsorbed on freshly cleaved mica for one minute before rinsing with milliQ water and blowing dry with nitrogen. A fresh FESP-V2 probe at a stiffness of 2.8N/m was utilized for all AFM imaging on an AFM Workshop Table-Top AFM

instrument. Observed events were then binned into five discrete categories by staff at the University of Michigan Single Molecule Analysis in Real-Time (SMART) Center, who were not aware of the biological significance of the samples. The categories used were: DNA only, Lrp only, Lrp-DNA complex (unbridged), Lrp-DNA complex with intramolecular bridging, and Lrp-DNA complex with intermolecular bridging.
