# Supplementary material for: Escherichia coli Leucine-Responsive Regulatory Protein Bridges DNA In Vivo and Tunably Dissociates in the Presence of Exogenous Leucine
Source: mBio. 2023 Feb 14;14(2):e02690-22. doi: 10.1128/mbio.02690-22 (PMC10127797; doi:10.1128/mbio.02690-22)
Supplement: TEXT S2 [file mbio.02690-22-s0003.pdf]

Text S2. Classes of Lrp-regulated genes

In considering the overall patterns of Lrp occupancy observed in Figure 2B, it is also useful to consider the clustering of Lrp binding regions (Y-axis of Fig. 2B), which separate into eight distinct classes (1-8). Class 1 consists of WT and D114E Min-Stat specific sites. Class 2 contains the D114E-Min specific binding sites, whereas Class 3 consists of regions bound by D114E regardless of the presence of leucine, demonstrating D114E's leucine insensitivity at only a subset of sites. Class 4 is a broad class of sites that are relatively leucine insensitively bound by WT, D114E, and L136R; these are likely some of the strongest Lrp binding sites since they remain occupied by Lrp even in LIV. Class 5 contains L136R-Stat specific sites, of which there are only a few. Conversely, the large Class 6 contains WT, D114E, and L136R Min specific peaks, as well as the L136R LIV peaks, which thus represent the 'normal' targets of Lrp in the minimal media condition and further demonstrate that L136R in LIV acts like WT in Min. Class 7 consists of predominantly L136R-Min and D114E-Min-Log peaks. Finally, Class 8 is made up of non-specific Lrp binding and could be attributed to noise in our peak-calling method.
