# Supplementary figures and images for: Escherichia coli Leucine-Responsive Regulatory Protein Bridges DNA In Vivo and Tunably Dissociates in the Presence of Exogenous Leucine
Source: mBio. 2023 Feb 14;14(2):e02690-22. doi: 10.1128/mbio.02690-22 (PMC10127797; doi:10.1128/mbio.02690-22)

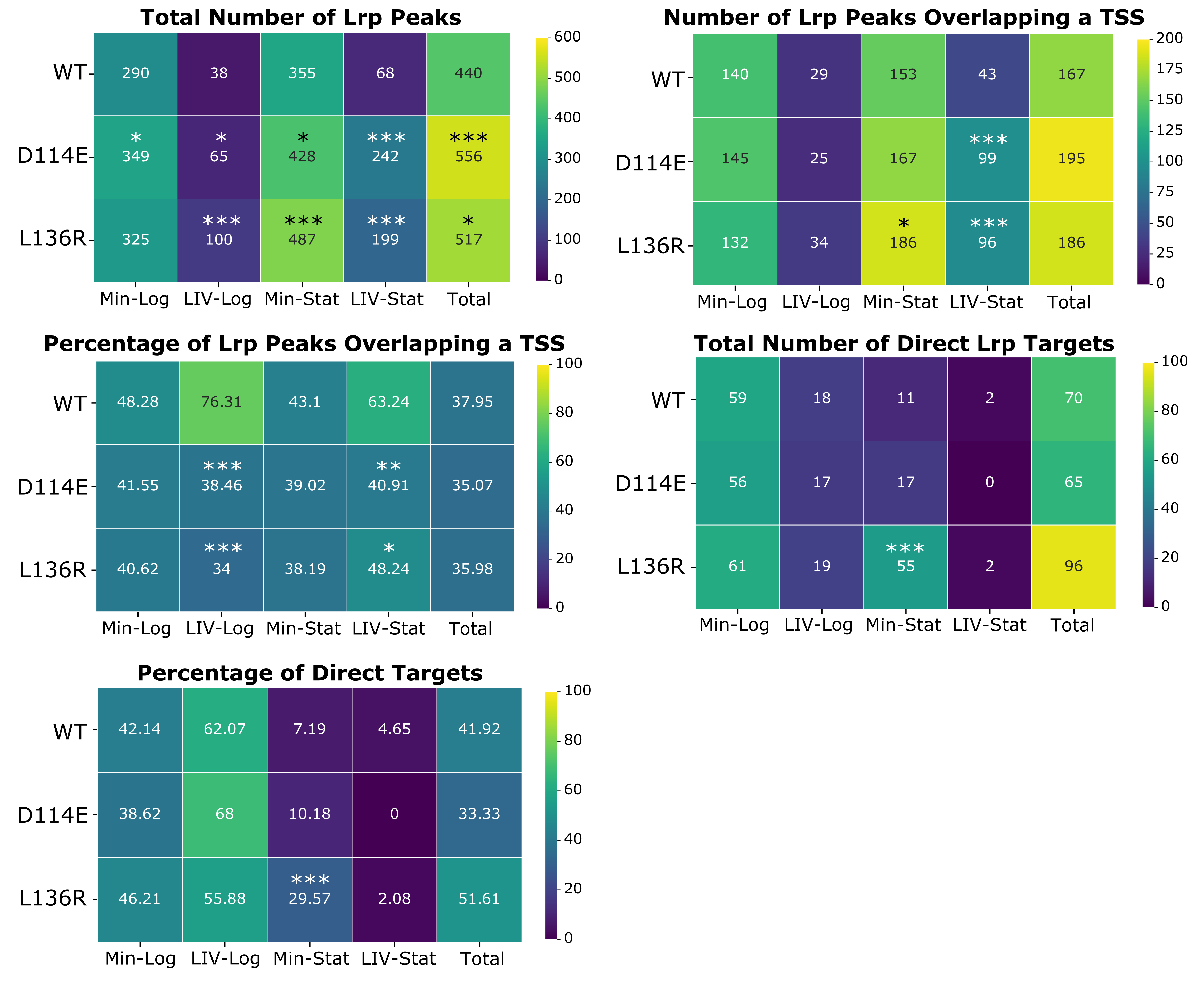

Supplement: FIG S1 [file mbio.02690-22-s0004.tif]

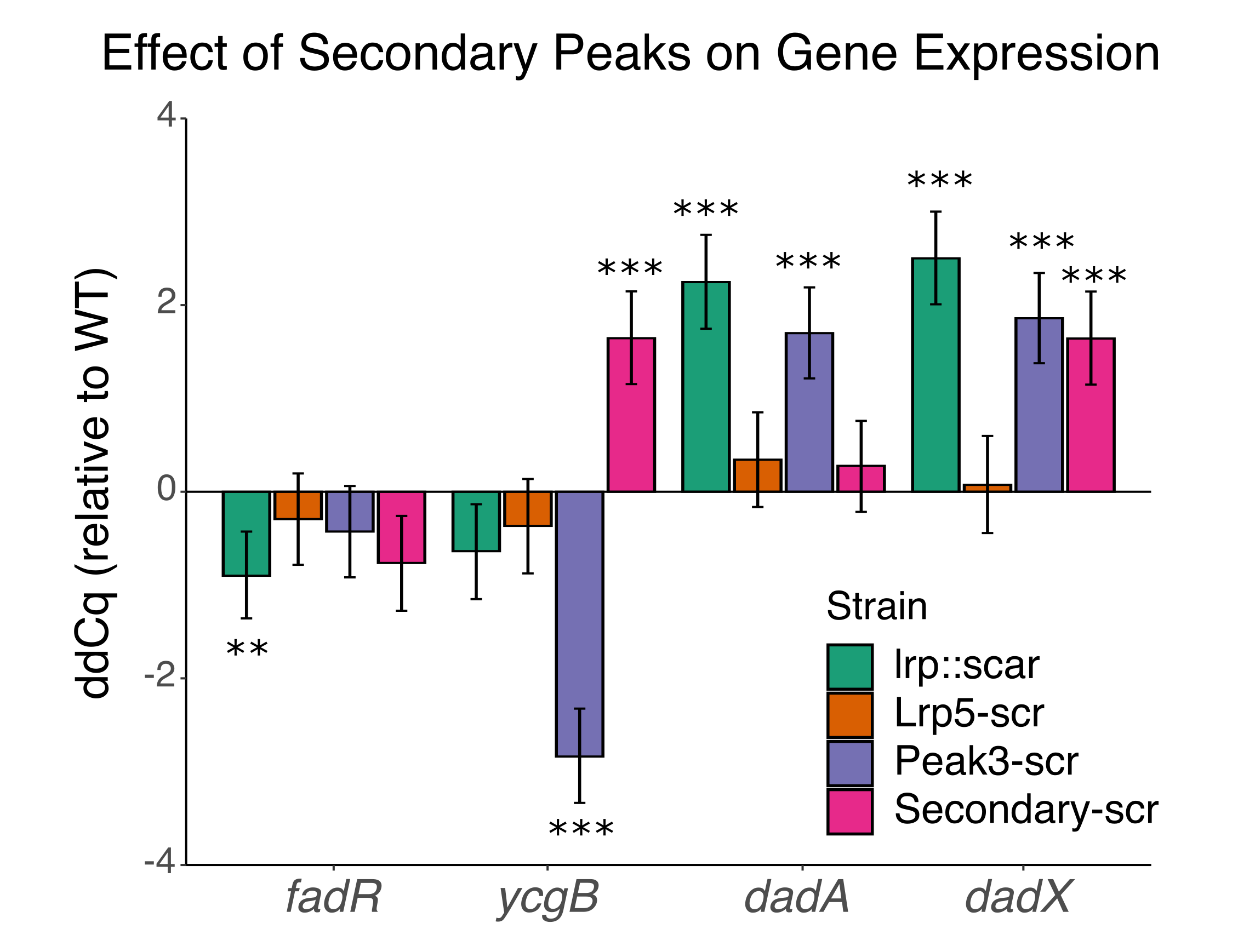

Supplement: FIG S2 [file mbio.02690-22-s0005.tif]

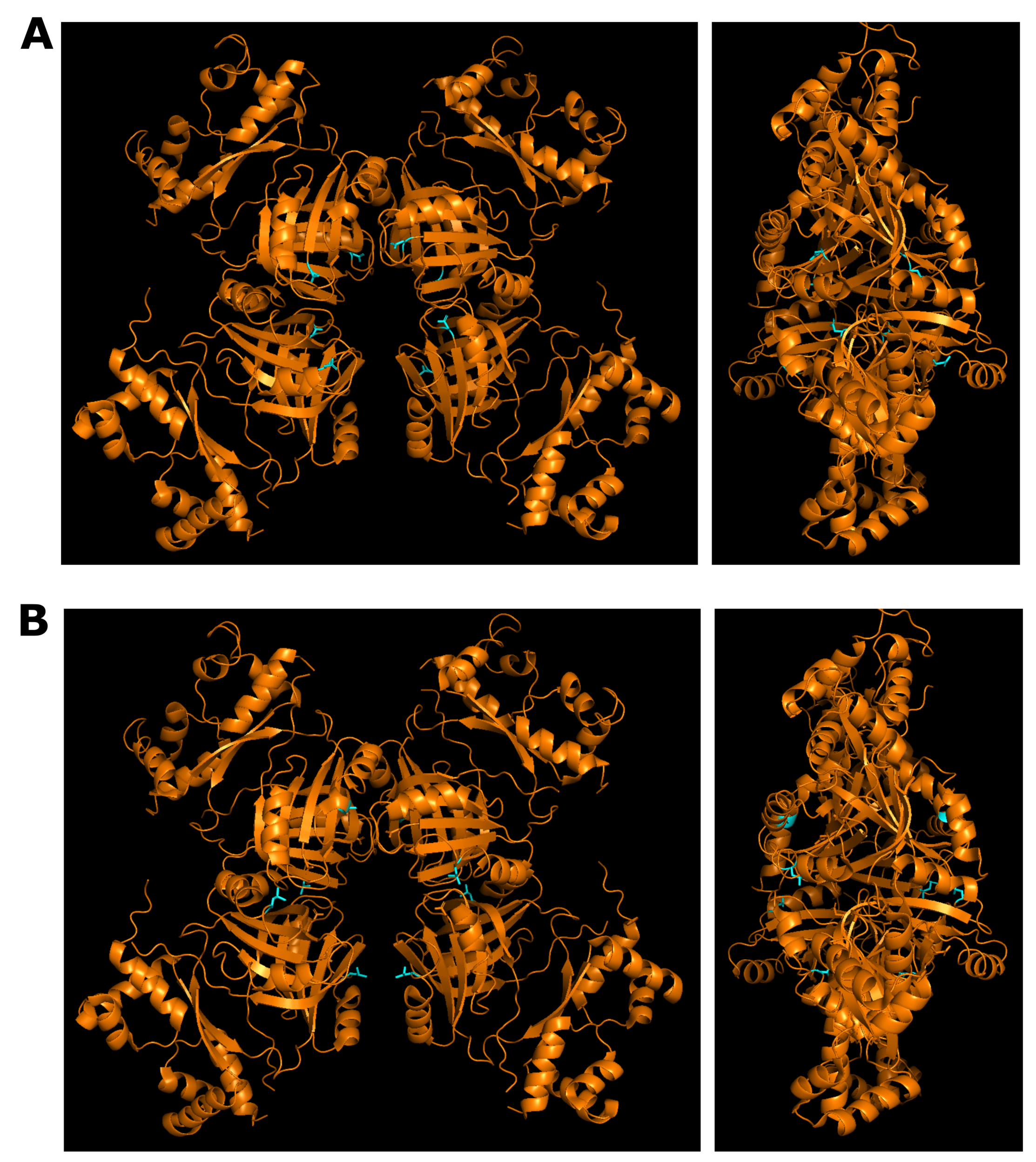

Supplement: FIG S3 [file mbio.02690-22-s0006.tif]
